# Development of CNS tropic AAV1-like variants with reduced liver-targeting following systemic administration in mice

**DOI:** 10.1016/j.ymthe.2024.01.024

**Published:** 2024-02-01

**Authors:** Matthieu Drouyer, Jessica Merjane, Deborah Nazareth, Maddison Knight, Suzanne Scott, Sophia H.Y. Liao, Samantha L. Ginn, Erhua Zhu, Ian E. Alexander, Leszek Lisowski

**Affiliations:** 1 Translational Vectorology Research Unit, Children’s Medical Research Institute, Faculty of Medicine and Health, The University of Sydney, Westmead, NSW, Australia; 2 Gene Therapy Research Unit, Children’s Medical Research Institute and Sydney Children’s Hospitals Network, Faculty of Medicine and Health, The University of Sydney, Westmead, NSW, Australia; 3 Discipline of Child and Adolescent Health, Faculty of Medicine and Health, The University of Sydney, Sydney, NSW, Australia; 4 Australian Genome Therapeutics Centre, Children’s Medical Research Institute and Sydney Children’s Hospitals Network, Westmead, NSW, Australia; 5 Laboratory of Molecular Oncology and Innovative Therapies, Military Institute of Medicine - National Research Institute, Warsaw, Poland

**Keywords:** AAV, directed evolution, rational engineering, adeno-associated virus, capsid, blood-brain barrier, liver detargeting, neutralizing antibody, LY6A

## Abstract

Directed evolution of natural AAV9 using peptide display libraries have been widely used in the search for an optimal recombinant AAV (rAAV) for transgene delivery across the blood-brain barrier (BBB) to the CNS following intravenous ( IV) injection. In this study, we used a different approach by creating a shuffled rAAV capsid library based on parental AAV serotypes 1 through 12. Following selection in mice, 3 novel variants closely related to AAV1, AAV-BBB6, AAV-BBB28, and AAV-BBB31, emerged as top candidates. In direct comparisons with AAV9, our novel variants demonstrated an over 270-fold improvement in CNS transduction and exhibited a clear bias toward neuronal cells. Intriguingly, our AAV-BBB variants relied on the LY6A cellular receptor for CNS entry, similar to AAV9 peptide variants AAV-PHP.eB and AAV.CAP-B10, despite the different bioengineering methods used and parental backgrounds. The variants also showed reduced transduction of both mouse liver and human primary hepatocytes *in vivo*. To increase clinical translatability, we enhanced the immune escape properties of our new variants by introducing additional modifications based on rational design. Overall, our study highlights the potential of AAV1-like vectors for efficient CNS transduction with reduced liver tropism, offering promising prospects for CNS gene therapies.

## Graphical abstract

**Figure:**
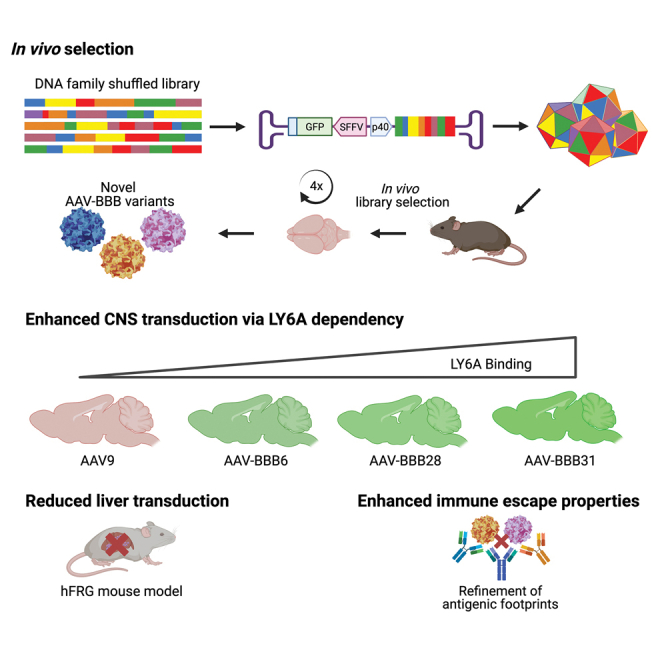

## Teaser

Lisowski and colleagues used a directed-evolution strategy in mice to develop novel rAAV variants capable of LY6A receptor-mediated crossing the blood-brain barrier (BBB) and with increased neuronal tropism after systemic administration. The AAV-BBB variants show reduced tropism for primary human hepatocytes *in vivo* compared to AAV9.

## Introduction

Vectors based on recombinant adeno-associated virus (rAAV) have become essential elements in the toolbox of gene therapy for the delivery of therapeutic genetic cargo to the target organs. The appeal of rAAVs lies in their nonpathogenic nature, ability to transduce both dividing and nondividing cells, and the permissibility to the capsid to undergo alterations at the residue level, allowing for the fine-tuning of the cellular tropism, transduction efficiency and immunological properties of the vector.^1^ However, the use of rAAVs for the treatment of CNS disorders is largely limited by the inability of current AAV capsids to efficiently cross the blood-brain barrier (BBB) following systemic administration.^2^

AAV9 is currently the most widely used vector in gene therapy trials targeting the CNS due to its ability to cross the BBB in small and large mammals, including humans.^3^^,^^4^ However, systemic injection for CNS targeting lacks specificity and efficiency, resulting in significant transduction of off-target tissues, with high risk of toxicity, particularly at high vector doses.^5^^,^^6^ To this end, significant side effects have been observed with the market-approved gene therapeutic Zolgensma, which uses AAV9 to deliver a functional copy of the *SMN1* gene for the treatment of spinal muscular atrophy (SMA). Although Zolgensma has had a revolutionary impact on the lives of SMA patients and their families, liver toxicity in patients has raised concerns about the safety of AAV9-based gene therapies,^7^^,^^8^ as well as rAAV-based therapies requiring systemic delivery in general. In addition, as wild-type (WT) AAVs are widely disseminated within the population, it is estimated that 30%–60% of the population harbor neutralizing antibodies (NAbs) against a range of naturally occurring AAVs, including AAV9.^9^ Unfortunately, patients positive for anti-AAV NAbs are excluded from clinical trials and may not be eligible for rAAV-based therapies, even after market approval. Considering this, and the lack of efficiency of current-generation rAAVs in gene delivery to the CNS, there is a critical need for novel improved rAAV vectors that are fit for this purpose. To maximize clinical impact, novel vectors for CNS delivery should possess improved efficiency in delivering therapeutic cargo to target cells, as well as the capability to evade recognition by preexisting anti-AAV NAbs. These characteristics will enable clinical efficacy at lower vector doses, increasing safety and reducing costs associated with novel therapies, while also making the therapies applicable to a larger proportion of the patients.

Because the receptors responsible for facilitating BBB crossing of AAVs are not well understood, directed evolution has emerged as a promising approach for the generation of novel AAV capsid variants capable of BBB crossing, because it does not depend on the understanding of the molecular mechanisms and receptors involved.^1^^,^^10^ Directed evolution involves subjecting large, highly variable libraries of rAAV variants to specific selective pressures, such as crossing the BBB and/or transduction of neurons, with the aim of identifying top candidates that exhibit desired characteristics. This method closely mimics natural evolution, but allows for the selection of novel traits in a controlled and accelerated manner. Because the outer capsid of the AAV is responsible for the majority of the sought-after properties, such as specific tissue tropism or immunological properties, it is the AAV *cap* gene that is commonly mutated for library generation. Historically, directed evolution approaches often focused on the key variable regions (VRs) contained within the AAV *cap* gene. These VRs are thought to be directly involved with receptor recognition and contribute to the unique characteristics of each AAV serotype.^11^ At these VR regions, short peptide libraries can be inserted to modify the interaction between the resulting AAV capsid and cellular receptors, as well as the immune system. Alternatively, libraries can be created that modify regions throughout the entire *cap* gene, using techniques such as error-prone PCR or capsid family shuffling.^1^ This strategy allows for the generation of novel AAV capsids with desired properties, such as enhanced BBB crossing and improved CNS transduction. Furthermore, it enables the identification of capsid signatures or motifs that directly contribute to CNS transduction, presenting a powerful genetic tool for overcoming existing knowledge gaps in gene therapy.

In this study, we applied the AAV capsid shuffle method to generate a library of diverse chimeric AAV capsid variants with the goal of identifying novel capsids with improved CNS tropism following systemic administration in mice. The library was generated by shuffling capsid gene sequences from AAV serotypes 1–12 (as previously described by our lab^11^) and candidate enrichment via 4 rounds of iterative selection for CNS tropic variants in mice following intravenous (IV) injection.

Using this approach, we identified 3 capsid variants, AAV-BBB6, AAV-BBB28, and AAV-BBB31, which exhibited >270-fold improvement in the functional transduction (FT) of the CNS following systemic administration in mice when compared to AAV9. It is interesting that the top capsids identified displayed a close relation to AAV1, a serotype isolated from nonhuman primates (NHPs) belonging to AAV clade A.^12^ In addition to improved CNS tropism, compared to AAV9, these vectors display a significant reduction in liver targeting *in vivo*, both in mouse hepatocytes and primary human hepatocytes in a chimeric human/mouse liver model. Through the implementation of rational design, we were able to further improve the immune escape properties of AAV-BBB6 and AAV-BBB28. Finally, it was determined through assessment in BALB/cJ mice and *in vitro* binding and transduction assays that the AAV-BBB variants displayed a dependence on LY6A binding for crossing the BBB in C57BL/6J mice. Thus, they use the same receptor responsible for the BBB crossing of AAV9 peptide variants AAV-PHP.eB and AAV.CAP-B10,^13^^,^^14^^,^^15^^,^^16^ despite that our new variants are based on a different parental capsid and were selected using a different method of directed evolution.

Overall, our study demonstrates the potential to use capsid shuffling and directed evolution to generate AAV capsids with improved CNS tropism following systemic administration based on non-AAV9 parental serotypes. The results of this study highlight 2 novel findings: (1) this study reports the first known instance of an identified *de novo* AAV-receptor interaction as a consequence of capsid shuffle bioengineering, and (2) we also present the first occurrence of 2 engineered capsid variants originating from distinct clades, yet using the same receptor to facilitate BBB crossing. Because the vast majority of BBB-penetrating capsids currently presented from the literature are AAV9 derived, the successful engineering of our AAV1 homolog variants present as valuable tools for gaining deeper insights into the mechanisms underpinning BBB crossing and harnessing the advantages of the unique properties associated with the diverse AAV clades in CNS capsid engineering.

## Results

### *In vivo* selection of shuffle capsids results in the enrichment of capsids closely related to AAV clade A

In the search for an AAV capsid with improved specificity and transduction efficiency in the CNS, we used a shuffled AAV capsid library based on parental variants AAV1–AAV12. In addition to the potential identification of highly functional novel AAV variants, this type of library can enable the identification of key capsid signatures, or fragments, important for CNS transduction.

**Figure 1:**
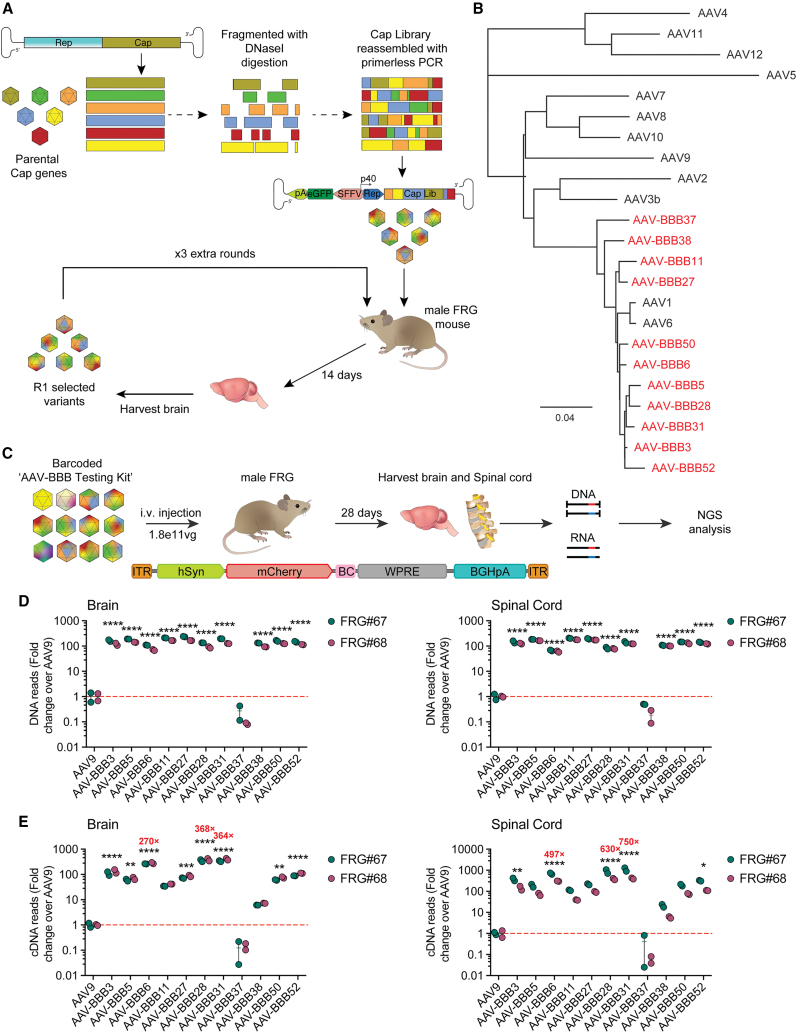
DNA family shuffling selection yields potent AAV-BBB capsid variants (A) Schematic workflow of DNA family shuffling and library selection. DNA family shuffling of AAV variants 1–12 was performed to create the library for evaluation, the AAV-BBB library. The AAV-BBB library was packaged into the FT-library platform^19^ and injected IV in male FRG mice. Four rounds of selection were performed in total. This selection method allows for the selection of highly enriched rAAV chimeric capsids in the mouse brain to be assessed at the level of DNA (cell entry) and cDNA (functional expression) using NGS analysis. (B) Phylogenetic tree of the AAV-BBB variants assessed compared to their parental capsids. (C) Parallel comparison of enriched AAV-BBB variants to AAV9. Following 4 rounds of library selection, 11 clones of the enriched mix were characterized and packaged for further analysis. These variants were packaged with an mCherry fluorescent marker driven by a neuron-restrictive promoter (hSyn) to highlight capsids with neuronal tropism. To allow the variants to be mixed and assessed in parallel, each capsid variant contained 2 unique genetic BCs. The 11 AAV-BBB variants were mixed at an equimolar ratio along with AAV9 (known as the AAV-BBB testing kit) and injected IV in 2 male FRG mice at a total dose of 1.8 × 10^11^ total vg. At 28 days post-injection, the brains and spinal cords of the mice were harvested and sent for NGS analysis at the DNA and cDNA level to determine the top-performing variants. (D and E) NGS analysis of the CNS tissues of the FRG mice following injection with the AAV-BBB testing kit at the (D) DNA level (cell entry) and (E) the cDNA level (functional expression). Data are represented as the mean ± SEM. ^∗^p ≤ 0.05; ^∗∗^p ≤ 0.01; ^∗∗∗^p ≤ 0.001; ^∗∗∗∗^p ≤ 0.0001. Statistical significance was calculated using a 1-way ANOVA with Dunnett’s multiple comparisons test, with AAV9 as the control.

The capsid library was cloned into our proprietary FT-library platform^17^ and packaged into AAV particles for the downstream assessment of capsid performance. The packaged AAV library was quantified using droplet digital PCR (ddPCR) and was injected into male naive Fah^−/−^/Rag2^−/−^/Il2rg^−/−^ (FRG)^18^ mice at a dose of 5 × 10^11^ vector genomes (vg) per animal using IV injection. The brain was harvested 3 weeks post-injection and genomic DNA (gDNA) was extracted. Following PCR-based recovery of the enriched capsid sequences, the PCR amplicon was used to clone the library into the naive FT recipient backbone to generate the secondary library (Figure 1A). In total, 4 consecutive rounds of selection were performed to allow for sufficient enrichment of AAV variants with enhanced FT ability in the CNS. Following the fourth round of selection, the capsid library was amplified from DNA and full-length capsid sequences were obtained from N = 37 random clones. The sequence analysis revealed the enrichment of a unique subpopulation of capsids closely related to the clade A AAV serotype family (Figures 1B, S1, and S2). Subsequently, N = 16 capsid variants (referred to as AAV-BBB variants) were selected for further evaluation based on diverse representation across the phylogram (Figure S1).

To perform functional evaluation of the selected capsids, each capsid coding sequence was cloned into a standard AAV packaging plasmid downstream of the AAV2 *rep* gene, with the AAV9 capsid additionally included for comparison. Each construct was subsequently used to package barcoded single-stranded (ss)AAV-hSyn-mCherry-BC-WPRE-BGHpA genomes using a standard iodixanol purification protocol. The barcode (BC) region allows for the identification and quantification of each capsid, facilitating robust parallel analysis of capsid performance to be assessed using next-generation sequencing (NGS). The use of 2 unique BCs per variant serves as an internal control.

Quantification analysis indicated that 5 of the selected AAV variants were inefficient at packaging and were thus excluded from further analysis. The remaining 11 variants were subsequently evaluated for their ability to transduce the CNS following IV administration in naive FRG mice. To enable direct side-by-side comparison, the 11 AAVs were mixed at an equimolar ratio to create an AAV-BBB testing kit similar to the strategy previously published by Westhaus et al.^17^ and injected IV at a total dose of 1.8 × 10^11^ vg per animal (equivalent to 1.5 × 10^10^ vg per variant) into 2 male FRG mice. At 28 days post-injection, the animals were sacrificed, with the brain and spinal cords harvested for analysis. Following DNA and RNA extraction and cDNA synthesis, the resulting nucleic acid was sent for NGS analysis to determine capsid performance at the cell entry (DNA) and expression (RNA) levels.

The analysis of the NGS reads revealed that 10 of the 11 assessed variants were able to transduce both the brain and the spinal cord at a significantly higher efficiency than the control AAV9, at both the DNA and RNA levels (Figures 1D and 1E). Among the assessed variants, AAV-BBB6, AAV-BBB28, and AAV-BBB31 emerged as the top performers, exhibiting a remarkable >270-fold and >500-fold improvement over AAV9 at the RNA level in the brain and spinal cord, respectively (Figure 1E). Consequently, these 3 vectors were selected for further analysis, including comprehensive characterization of vector efficiency in the mouse CNS, as well as analysis of their abilities to target peripheral tissues.

### AAV-BBB28 and AAV-BBB31 demonstrate CNS transduction in mice with significant liver detargeting when compared to AAV9 and parental serotype AAV1

Before conducting the *in vivo* evaluation, we performed an analysis of the vector packaging efficiency of AAV-BBB6, AAV-BBB28, and AAV-BBB31 in comparison to AAV9 (Figure S3A). Although the packaging efficiency of AAV-BBB28 was lower than AAV9, 2 new variants, AAV-BBB6 and AAV-BBB31, packaged with similar efficiency to AAV9.

Detailed sequence analysis revealed that AAV-BBB6, AAV-BBB28, and AAV-BBB31 differed from their closest parental serotype, AAV1, by only 12, 20, and 22 amino acids, respectively (Figure S13). Modeling of the VP3 monomers of the AAV-BBB variants revealed that structural differences evident only within the surface exposed the VR-V loop when compared to AAV1 (Figures S3B and S3C). It was interesting that there were only 2 mutations within the VR-V region that were present in all 3 AAV-BBB variants: deletion of the asparagine (N) at position 498 (N498) and a threonine-to-alanine substitution at position 502 (T502A). Given this information, we hypothesized that these 2 mutations were responsible for the observed structural change in VR-V and that they likely had a significant impact on the function of the AAV-BBB variants because of their conservation across the top-performing AAV-BBB variants. It has been shown previously that T502 is an important residue for AAV1 interaction with sialic acid,^20^ and A502 is a required AAV9 residue for both galactose binding and post-attachment viral processing.^21^ To determine whether the 2 changes seen in the AAV-BBB variants affected their ability to bind sialic acid, we took advantage of 2 types of Chinese hamster ovary (CHO) cell lines: Pro5 and Lec2. Specifically, Pro5 lines contain surface-exposed sialic acid residues, whereas Lec2 cells have exposed surface glycans due to mutation in their sialic acid transporter. Because of the surface-exposed sialic acid residues, the CHO-Pro5 line facilitates the binding of AAVs that rely on sialic acid binding, such as AAV1, and the CHO-Lec2 line facilitates the binding of galactose residue binding vectors, such as AAV9. All 3 AAV-BBB variants showed the same reduced level of cell binding and transgene expression in the Lec2 and Pro5 cells, as measured by the number of vector genomes bound to the cell (Figure S3D), and the percentage of EGFP^+^ cells quantified via fluorescence-activated cell sorting (FACS) (Figure S3E). This finding suggests that the specific capsid changes present in our AAV-BBB variants abolished the binding to sialic acid and did not facilitate binding to terminal galactose. This indicates that an alternative receptor is used by the AAV-BBB variants to bind to and enter the target cells.

To assess the individual performance of the novel AAV-BBB variants in comparison to AAV9 and AAV1 across various murine tissues, the variant capsids were used to package a ssAAV cassette encoding an mCherry fluorescent protein under the control of the cytomegalovirus (CMV) promoter (ssAAV-CMV-mCherry-WPRE-BGHpA). The ubiquitous CMV promoter was chosen in contrast to the previously used hSyn promoter (Figure 1C) to allow us to evaluate the expression of the novel variants in the CNS and other tissues.

Following vector production, we examined the brain tropisms of the novel AAV-BBB variants in adult male FRG mice (C57BL/6J background). Individual variants were injected into mice at a dose of 5 × 10^11^ vg per animal, with N = 3 animals per vector. Mice were harvested 3 weeks post-injection, with their tissues harvested for further analysis of AAV efficiency, tropism, and biodistribution (including brain, spinal cord, liver, heart, muscle, and kidney).

**Figure 2:**
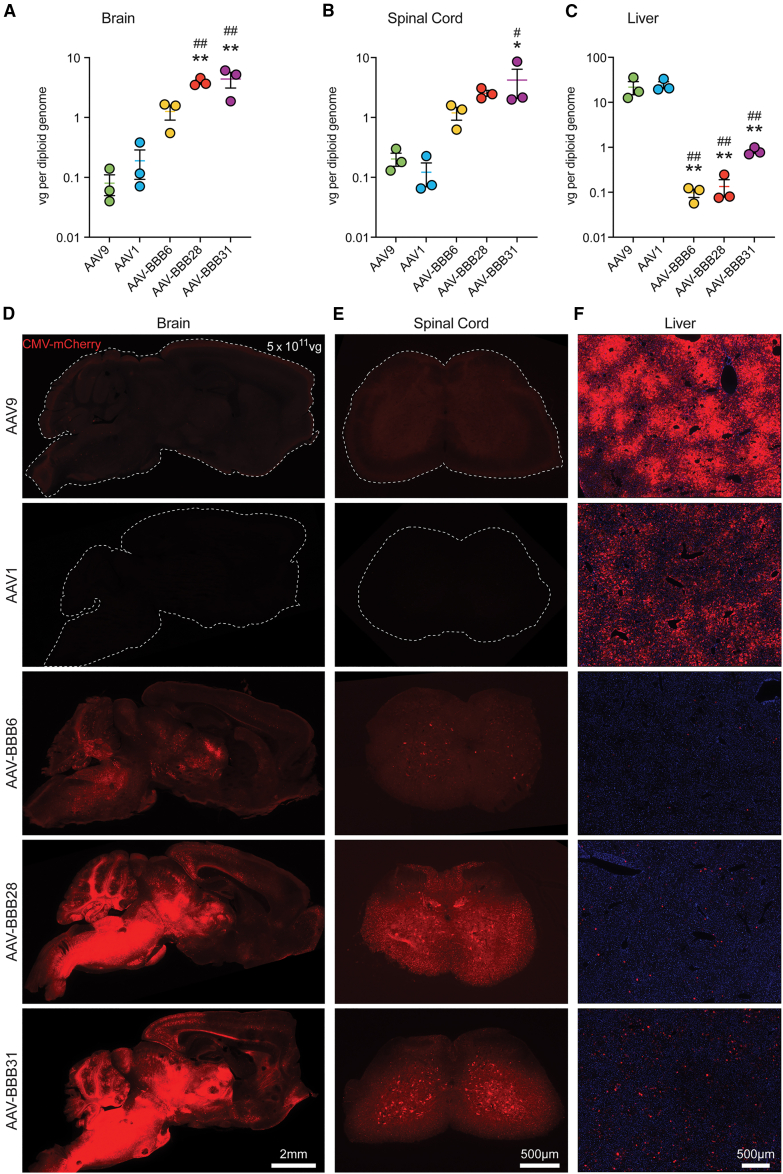
CNS transduction profile of novel AAV-BBB variants compared with AAV9 Individual comparison of top-performing AAV-BBB variants to AAV9. The capsid variants AAV9, AAV-BBB6, AAV-BBB28, and AAV-BBB31 were packaged with a transgene containing an mCherry reporter under the control of the ubiquitous CMV promoter. Each individual capsid variant was IV administered into male FRG mice (N = 3, dose: 5 × 10^11^ total vg per animal). Mouse tissues were harvested 3 weeks post-injection. VCN was assessed in the (A) brain, (B) spinal cord, and (C) liver and represented by vector genome per diploid cell (normalized to mouse actin-β). Data are represented as the mean ± SEM. Statistical significance was calculated using a 1-way ANOVA with Dunnett’s multiple comparisons test; ^∗^p ≤ 0.05, ^∗∗^p ≤ 0.01 versus AAV9; or ^#^p ≤ 0.05, ^##^p ≤ 0.01 versus AAV1. Transgene expression was also assessed by mCherry fluorescence in the (D) brain, (E) spinal cord, and (F) liver. Scale bars: 2 mm (brain); 500 μm (spinal cord and liver).

Compared to AAV9, we observed a significant increase in vector copy number (VCN) in mouse brain for AAV-BBB28 and AAV-BBB31, with a 49-fold and 55-fold increase, respectively (Figures 2A and 2B). Although not statistically significant, noticeable increases in VCN were also observed in the spinal cord for all of the variants. Notably, all of the vectors exhibited significant detargeting from murine liver, with the liver VCN of AAV-BBB6, AAV-BBB28, and AAV-BBB31 measured to be 224-fold, 162-fold, and 27-fold lower than AAV9, respectively (Figure 2C). It is interesting to note that the significantly lower liver transduction from our AAV-BBB variants differs from the parental AAV1 capsid as measured with VCN (Figure 2C).

**Figure 3:**
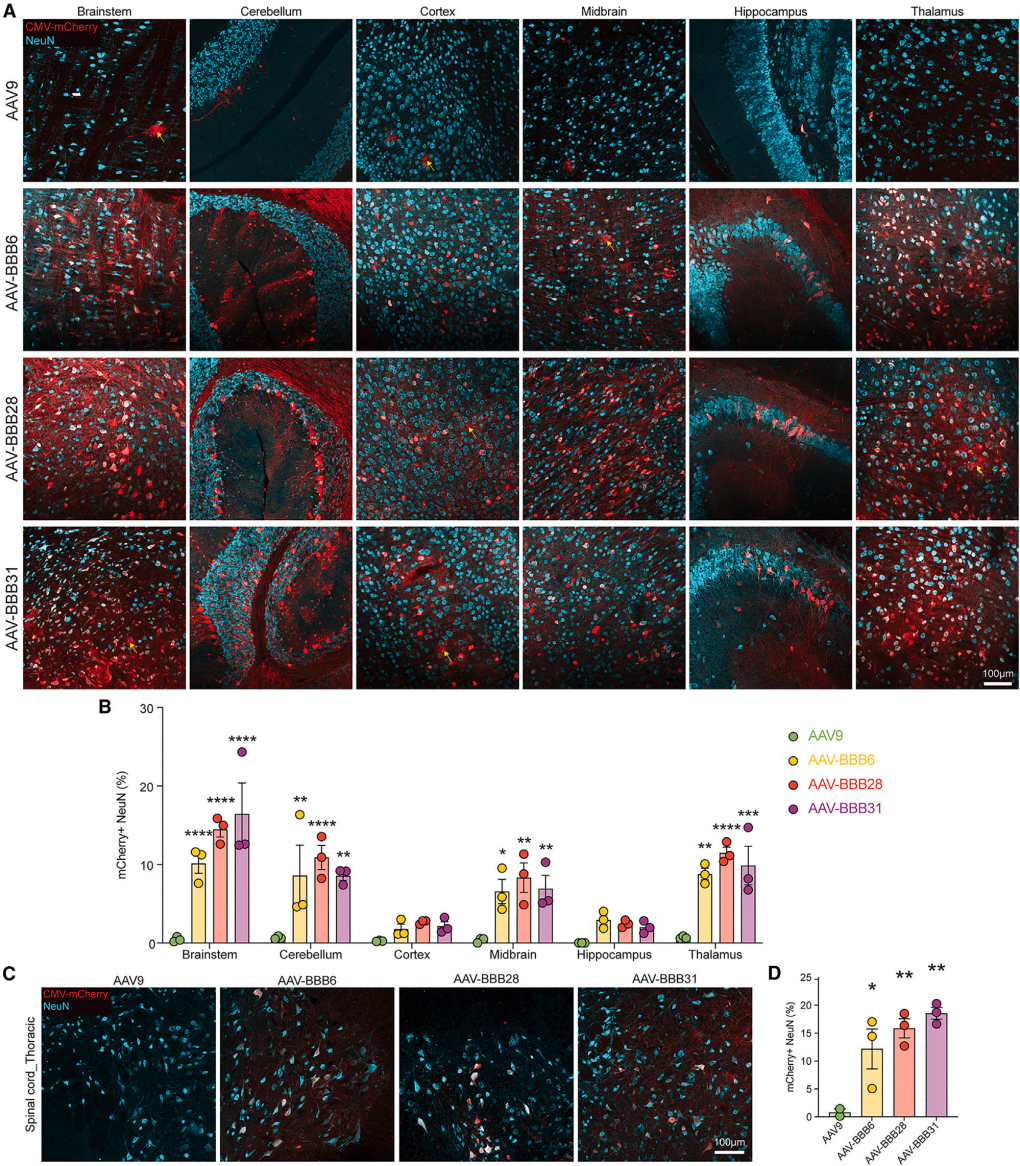
AAV-BBB variants favor neuronal transduction Following individual injection of the AAV-BBB variants and AAV9 IV in FRG mice (N = 3, dose: 5 × 10^11^ total vg per animal), tissues were harvested 3 weeks post-injection for downstream analysis. (A) Immunohistochemical analysis of mouse brain sagittal sections stained with NeuN to identify neurons (cyan). Representative images of different CNS regions—brainstem, cerebellum, cortex, midbrain, hippocampus, and thalamus—are shown. Yellow arrows indicate examples of mCherry^+^ astrocytes identified by morphology. (B) Percentage of mCherry^+^ neurons in the brainstem, cerebellum, cortex, midbrain, hippocampus, and thalamus of mice injected with AAV9 or AAV-BBB variants. Data are represented as the mean ± SEM. Individual data points represent the average of 3–5 nonoverlapping images per region taken with a 20× objective.^∗^p ≤ 0.05; ^∗∗^p ≤ 0.01; ^∗∗∗^p ≤ 0.001; ^∗∗∗∗^p ≤ 0.0001. Statistical significance was calculated using a 2-way ANOVA with Dunnett’s multiple comparisons test, with AAV9 as the control. (C) IHC analysis of mouse spinal cord (thoracic) sections stained with NeuN to identify neurons (cyan). Scale bars: 100 μm (D) Percentage of mCherry^+^ neurons in the spinal cord of mice injected with AAV9 or AAV-BBB variants. Data are represented as the mean ± SEM. ^∗^p ≤ 0.05; ^∗∗^p ≤ 0.01. Statistical significance was calculated using a 1-way ANOVA with Dunnett’s multiple comparisons test, with AAV9 as the control.

The trends observed for the VCN aligned with the pattern of mCherry expression throughout the brains, spinal cords, and livers of the treated mice (Figures 2D–2F). In the brain and spinal cord, the greatest expression was observed for AAV-BBB31, followed by AAV-BBB28 and AAV-BBB6 (Figures 2D and 2E). The regions with the highest tropism appeared to be the brainstem, cerebellum, midbrain, and thalamus (Figures 3A and 3B). Quantitative analysis of mCherry^+^ neurons revealed a significant increase across all regions, with each AAV-BBB variant compared to AAV9, except for the cortex and hippocampus (Figure 3B).

To further elucidate the cellular tropism of the AAV-BBB variants, we examined the colocalization of vector-encoded mCherry with glial fibrillary acidic protein (GFAP) (astrocytes) and olig2 (oligodendrocytes), revealing minimal overlap (Figure S4). In contrast, the AAV-BBB variants exhibited enhanced targeting of Purkinje cells in the cerebellum, with AAV-BBB28 demonstrating a significant increase in mCherry^+^ Purkinje cells compared to AAV9. These findings collectively support the neuronal preference of our AAV-BBB variants, as indicated by mCherry colocalization with NeuN (Figure 3) and calbindin (Figure S5).

To assess the biodistribution of the vectors in other peripheral tissues, we examined the VCN and mCherry expression in the hearts, muscles, and kidneys (Figure S6). In the heart and muscle, no significant differences in VCN or mCherry fluorescence intensity were observed when compared to AAV9. Upon VCN analysis in the kidney, we found a significant increase in vector DNA for AAV-BBB6 and AAV-BBB31 variants compared to AAV9. However, it is worth noting that the expression of the mCherry reporter at the protein level for all of the AAV-BBB variants was virtually undetectable in the kidney (Figure S6C). This result suggests that all 3 AAV-BBB variants can enter the kidney with high efficiency but are unable to functionally express their encoded transgene. This discrepancy between AAV entry and transgene expression in the kidney indicates incomplete intracellular trafficking.

To more closely evaluate the function of the AAV-BBB vectors in the CNS, the top-performing AAV-BBB variants, AAV-BBB28 and AAV-BBB31, along with AAV9 were used to package a reporter cassette encoding mCherry reporter under the control of a neuron-restrictive hSyn promoter. The vectors were injected systemically (IV) into male naive FRG mice at a dose of 5 × 10^11^ vg per animal (N = 2 animals per vector) and harvested 3 weeks post-injection. Although the AAV-BBB variants continued to exhibit a preference for neuronal transduction using the ubiquitous CMV promoter, the switch to the hSyn promoter facilitated a more widespread transduction, leading to the observation of transduced neurons throughout the cortex (Figures S7A and S7B). This expanded transduction pattern was not previously observed with the CMV promoter (Figure 2). Quantification of the number of mCherry^+^ cells colocalized with NeuN in the cortex supported this observation, increasing significantly with the use of the hSyn promoter (2.5-fold and 4-fold improvement over the CMV promoter for AAV-BBB28 and AAV-BBB31, respectively) (Figure S7C).

### AAV-BBB variants demonstrate reduced targeting of human hepatocytes in a hFRG chimeric liver *in vivo* model compared to AAV9

Although the VCN and mCherry expression data confirm that our novel variants were significantly detargeted from the mouse liver, it was important to study whether the same effect would be observed for human liver because vector tropism can be inconsistent across species.^22^^,^^23^ Therefore, to determine whether our novel variants were also detargeted from human primary hepatocytes, female FRG mice were engrafted with primary human hepatocytes to generate a well-established *in vivo* xenograft model of human liver.^22^ Humanized FRG (hFRG) mice were individually injected with the top 2 performing variants, AAV-BBB28 and AAV-BBB31, encoding ssAAV-CMV-EGFP transgene cassettes. AAV9 and AAV1 encoding the same cassette were included as controls. Mice were injected IV at a dose of 5 × 10^11^ vg per animal (N = 2 animals per vector) and harvested 3 weeks post-vector administration. The mouse brain and spinal cord were harvested for immunohistology and VCN analysis (Figure S8). For the chimeric liver, 1 lobe was isolated for immunohistology analysis before liver perfusion. The remaining liver was used to isolate the human hepatocyte population using FACS. The isolated human hepatocytes were subsequently used for VCN analysis.

**Figure 4:**
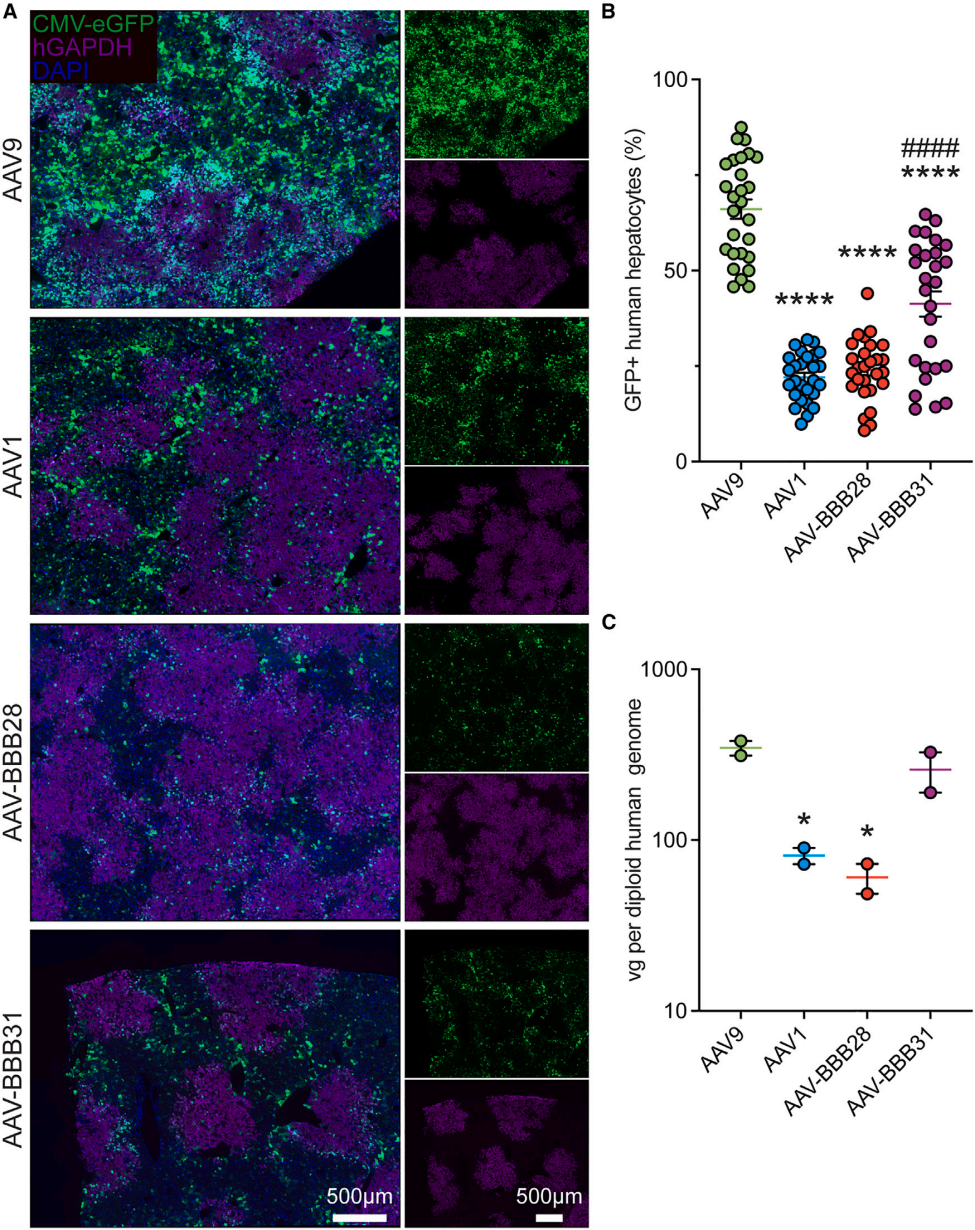
AAV-BBB variants retain liver-detargeting properties from parental AAV1 in hFRG mice when compared to AAV9 Individual comparison of top-performing AAV-BBB variants to AAV9. The capsid variants AAV9, AAV-BBB28, and AAV-BBB31 were packaged with a transgene containing a GFP reporter under the control of the ubiquitous CMV promoter. Each individual capsid variant was IV administered into N = 2 hFRG mice (dose: 5 × 10^11^ total vg per animal). Mice tissues were harvested 4 weeks post-injection, with 1 lobe of the chimeric mouse/human liver collected for IHC analysis. The remaining liver was used to isolate the human hepatocyte population using FACS for analysis of the VCN. (A) Transduced cells are indicated by GFP expression (green). Human clusters are shown by human GAPDH staining (purple). Scale bars: 500 μm. (B) Percentage of GFP^+^ human hepatocyte cells (N = 12–15 human clusters per mouse were analyzed). (C) VCN analysis of human hepatocytes isolated using FACS. Data are represented as the mean ± SEM. Statistical significance was calculated using a 1-way ANOVA with Dunnett’s multiple comparisons test; ^∗^p ≤ 0.05, ^∗∗∗∗^p ≤ 0.0001 versus AAV9, or ^####^p ≤ 0.0001 versus AAV1.

Consistent with the results obtained in the nonengrafted naive male FRG mice, AAV-BBB28 and AAV-BBB31 demonstrated a visible reduction in EGFP expression in the chimeric liver when compared to both AAV9 and parental AAV1 (Figure 4A). For the BBB variants, the low level of EGFP expression present appeared to primarily colocalize with the mouse hepatocytes, as evidenced by the fact that EGFP^+^ cells appeared to be negative for the human glyceraldehyde 3-phosphate dehydrogenase (GAPDH) marker (Figure 4A). This conclusion was further supported by the significant reduction in the percentage of EGFP^+^ cells per human cluster (Figure 4B). VCN analysis revealed that although AAV-BBB31 has lower average VCN per human diploid genome, the difference was not statistically significant compared AAV9. However, AAV-BBB28 showed a significantly lower average VCN in human cells (Figure 4C), which is consistent with the percentage of EGFP^+^ human cells (Figure 4B), as compared with AAV9. Importantly, AAV1 also displayed a significantly lower human liver transduction compared to AAV9, suggesting that AAV-BBB28 and AAV-BBB31 retain the naturally reduced liver tropism properties observed in the parental AAV1 capsid (Figure 4).

Consistent with data obtained in the naive FRGs, AAV-BBB28 and AAV-BBB31 demonstrated stronger expression than AAV9 in the brain and the spinal cord. However, the VCNs were reduced compared to the nonengrafted male FRG mice receiving the same total vector dose IV (Figure S8). These differences may be attributed to the previously observed discrepancies in transduction efficiency between female and male C57BL/6J mice.^24^ Another possible explanation is that the higher VCN in the hFRG liver compared to the naive FRG liver may result in partial sequestration of the AAV, reducing the amount of free circulating AAVs in the blood available for BBB crossing. Although we are currently unable to validate or disprove either of these hypotheses, it is also possible that the observed effect is a net result of both mechanisms.

### Rational refinement of antigenic footprints results in improved intravenous immunoglobulin (IVIg) resistance

**Figure 5:**
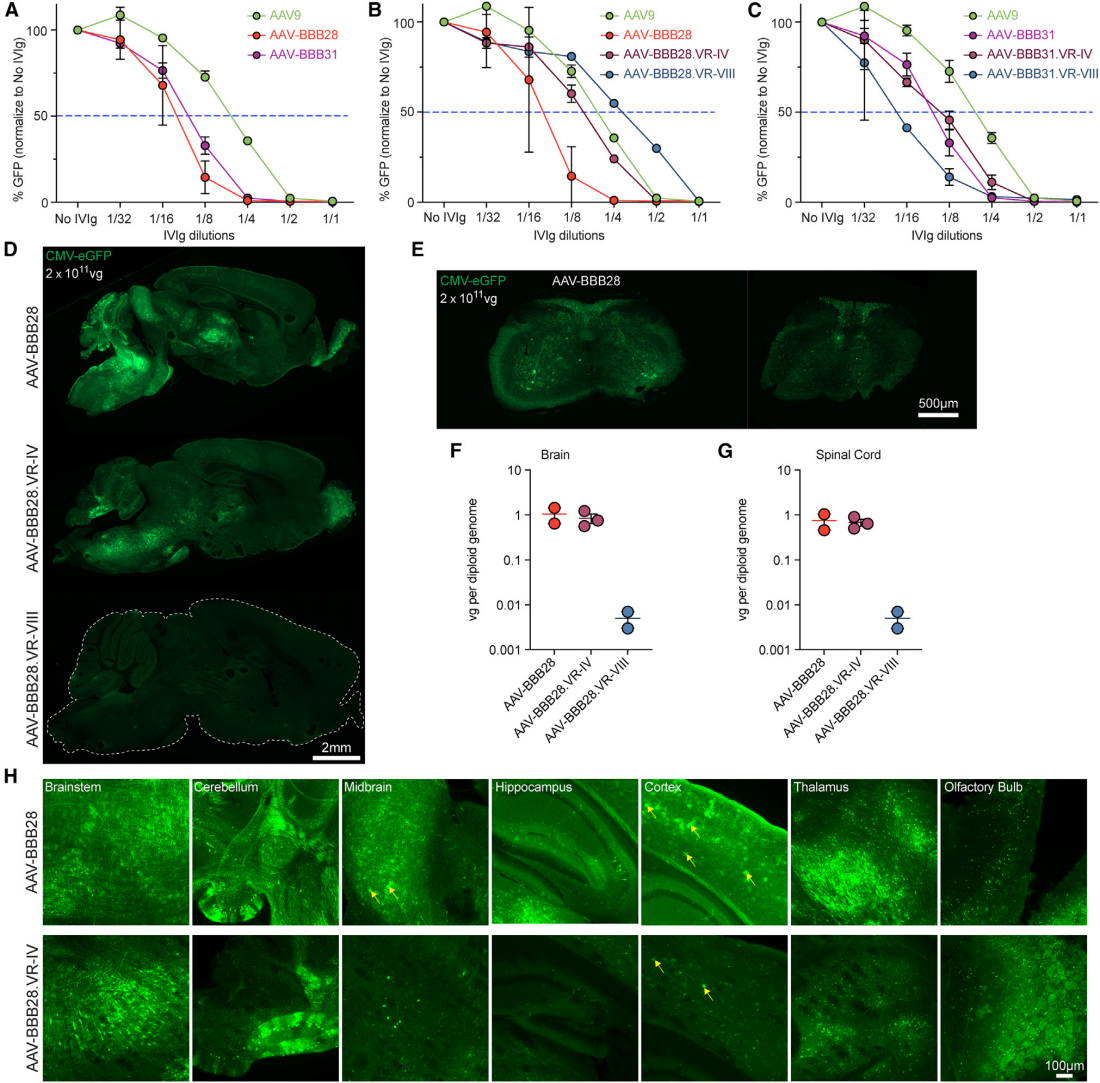
Generation of AAV-BBB escape variants via rational capsid engineering (A–C) Neutralization assay of (A) WT AAV-BBB vectors, (B) immune escape variants of AAV-BBB28 and (C) immune escape variants of AAV-BBB31 following preincubation with human IVIg before transduction of HEK293T cells. The percentage of GFP^+^ cells 48 h after transduction was analyzed by flow cytometry (N = 3 independent experiments). The dotted blue line represents IVIg-mediated inhibition of AAV transduction by 50%. (D–H) CNS transduction profile of AAV-BBB28.VR-IV (N = 3) and AAV-BBB28.VR-VIII (N = 2) in FRG mouse CNS as compared with parental AAV-BBB28 (N = 2). Eight-week-old FRG mice were injected IV with a dose of 2 × 10^11^ total vg per animal. Mice were harvested 3 weeks post-injection for IHC and VCN analysis. (D and E) GFP expression (green) of AAV-BBB28 variants in the FRG mouse (D) brain and (E) spinal cord. Scale bars: 2 mm (brain); 500 μm (spinal cord). The VCN of each variant is indicated for both (F) brain and (G) spinal cord. Data are represented as the mean ± SEM. (H) Region-specific GFP expression in the brain. Yellow arrows indicate examples of GFP^+^ astrocytes identified by morphology. Scale bar: 100 μm.

Because pre-existing immunity against AAV remains one of the major challenges in clinical gene therapy application,^25^ we assessed our novel AAV-BBB variants for their ability to resist neutralization by human pooled IVIg. To do so, we performed an *in vitro* AAV neutralization assay on HEK293T cells at increasing concentrations of IVIg, using AAV9 as a control (Figures 5A–5C). Notably, our results revealed that our novel AAV-BBB variants exhibited decreased resistance to IVIg neutralization when compared to AAV9 (Figure 5A). The data showed that the concentration of IVIg required to achieve a 50% decrease in transduction (half-maximal inhibitory concentration) was 2-fold lower for all AAV-BBB variants in comparison to AAV9 (∼1/8 for AAV-BBB variants vs. 1/4 for AAV9) (Figure 5A). This finding correlates to a lower concentration of IVIg required to inhibit cellular transduction of our AAV-BBB variants compared to AAV9.

To bypass the immune system to retain the safety and efficacy of AAV vectors, Tse et al. mapped key regions on AAV1 that are responsible for capsid interaction with the NAbs.^26^ Because our variants are highly homologous to AAV1, we hypothesized that by altering the antigenic footprints of the capsid, we could bypass the recognition by the immune system while retaining the functional properties of our AAV vectors. To this end, we modified the 2 key antigenic sites identified by Tse et al. in our novel AAV variants with the aim of increasing their immune escape properties. Specifically, amino acid residues within 2 surface regions—VR-IV (456-AQNK-459) and VR-VIII (588-STDPATGDVH-597)—were replaced with (456-SERR-459) and (588-DLDPKATEVE-597), respectively.^26^

In the case of AAV-BBB28, the addition of the VR-IV or VR-VIII mutation improved the resistance to the IVIg, with the inclusion of the VR-VIII mutation having the most effect (Figures 5B and 5C). It is interesting that in the context of AAV-BBB31, the VR-IV mutation had no effect on IVIg resistance, whereas the VR-VIII mutation resulted in a decreased resistance to IVIg neutralization compared to AAV-BBB31 (Figure 5C). We subsequently evaluated the prevalence of NAbs against the novel AAV-BBB variants using a small cohort of individual human sera (N = 8), but we were not able to find any significant difference in seroprevalence between the AAV-BBB variants and the control AAV9 (Figure S9). Interestingly, while AAV-BBB28.VR-IV showed an increased ability to escape NAbs (Figure 5B), the VR mutant displayed a seroprevalence comparable to AAV9, and AAV-BBB28 (Figure S9).

To assess whether the immune escape variants of AAV-BBB28 retained the ability to cross the BBB *in vivo* following the capsid modifications, AAV-BBB28, AAV-BBB28.VR-IV, and AAV-BBB28.VR-VIII variants were injected IV at a dose of 2 × 10^11^ vg per animal into naive male FRG mice (Figures 5D–5H). Although analysis of VCN in the brain indicated no significant decrease in the ability of the vector to cross the BBB between AAV-BBB28 and AAV-BBB28.VR-IV, the ability of AAV-BBB28.VR-VIII to cross the BBB appeared to be abolished (Figures 5D–5G). Despite the capsid modification having no impact on the ability of AAV-BBB28.VR-IV to enter cells, the immunohistochemistry (IHC) analysis indicated a reduction in transgene expression from the AAV-BBB28.VR-IV variant when compared to AAV-BBB28 (Figures 5D and 5E). This reduction in expression was particularly noticeable when observing the number of GFP^+^ cells in the midbrain, hippocampus, and cortex region (Figure 5H).

Of note, the preliminary assessment of AAV-BBB6 with its AAV-BBB6.VR-IV and AAV-BBB6.VR-VIII variants revealed the same trend as AAV-BBB28, showing an increase in immune escape, although this resulted in a reduction in brain transduction with AAV-BBB6.VR-IV and a complete impairment of BBB crossing for AAV-BBB6.VR-VIII (Figure S10).

### Novel AAV-BBB variants fail to cross the BBB in BALB/cJ mice due to LY6A dependence

Previously published studies reported that the ability of AAV to traverse the BBB may differ between mouse strains. One such example is the AAV9 variant PHP.B, which is able to traverse the BBB in C57BL/6J mice but not BALB/cJ.^14^^,^^15^^,^^16^

To evaluate the BBB crossing efficiency of our AAV-BBB variants in BALB/cJ mice, we injected AAV-BBB28 and AAV9 as a control IV into BALB/cJ mice. Although improvement in IVIg escape could not be achieved previously with AAV-BBB31, due to its superior transduction ability, it was also included in this analysis. Each variant was administered at a dose of 5 × 10^11^ vg per animal, and brain, spinal cord, and liver tissues were collected for analysis 3 weeks post-injection.

**Figure 6:**
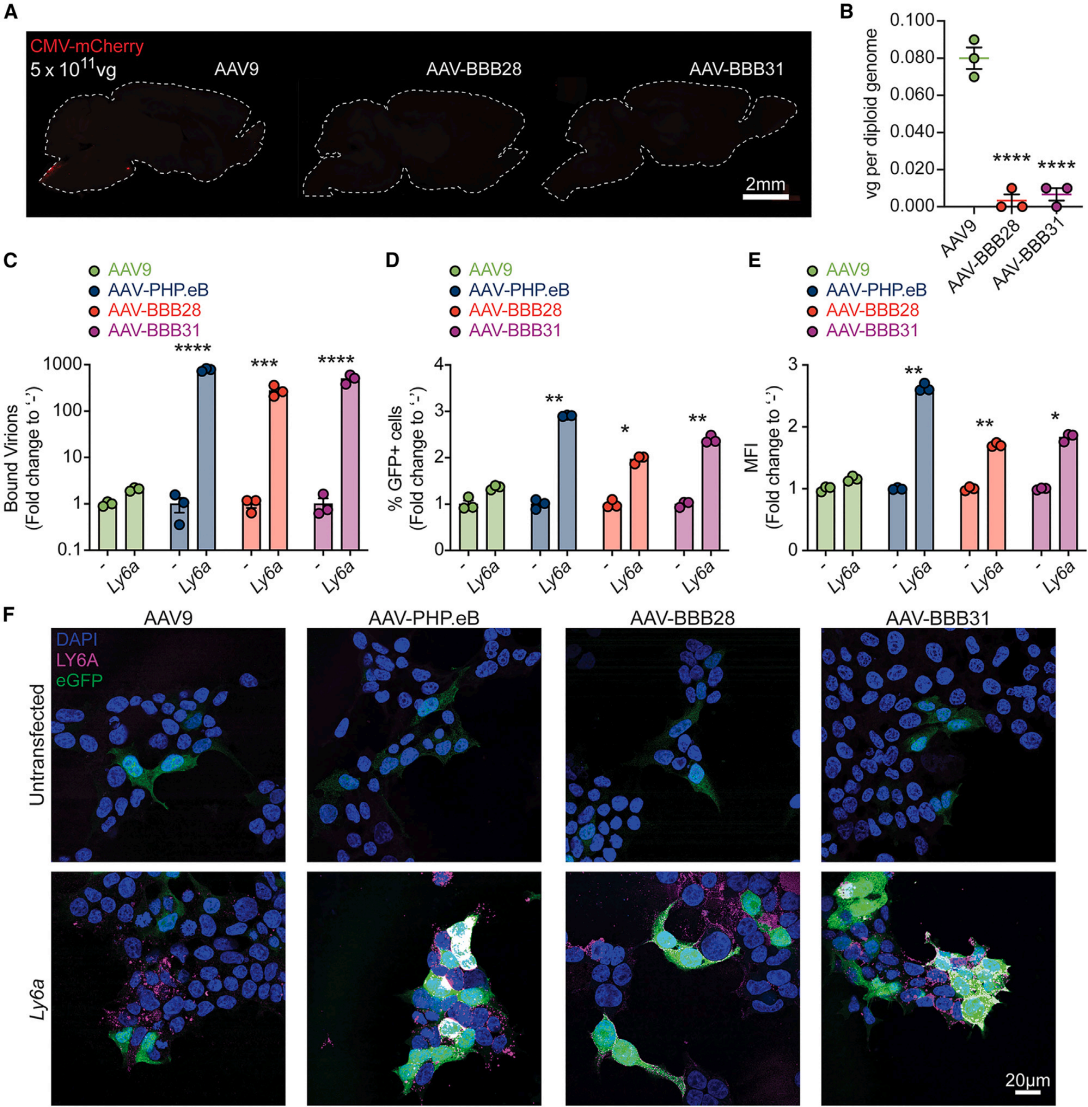
Brain transduction of AAV-BBB variants demonstrate LY6A dependence (A) IHC comparison of AAV vector performance following IV injection of AAV-BBB28 or AAV-BBB31 compared to AAV9 in BALB/cJ mice. All of the variants were used to package a transgene cassette encoding an mCherry reporter under the control of the ubiquitous CMV promoter and injected at a dose of 5 × 10^11^ total vg per animal. Transgene expression was assessed by mCherry fluorescence in the brain. Scale bar: 2 mm. (B) VCN analysis of the BALB/cJ mouse brains following IV injection as described in (A). VCN was determined by ddPCR of the viral genomes. Data are shown as mean ± SEM. N = 3. ^∗∗∗∗^p ≤ 0.0001. Statistical significance was calculated using a 1-way ANOVA with Dunnett’s multiple comparisons test, with AAV9 as the control. (C) Cell binding assay of AAV9, AAV-PHP.eB, AAV-BBB28, and AAV-BBB31 in HEK293T transfected with *Ly6a* or untransfected control. Binding was assessed by ddPCR of the viral genome. Data are shown as mean ± SEM. N = 3. (D and E) Transduction assay of aforementioned AAV variants as measured by FACS 48 h post-transduction. Transduction efficiency is measured by (D) percentage of GFP^+^ cells and (E) MFI quantification. Data are shown as mean ± SEM. N = 3. (F) Native GFP fluorescence in HEK293T cells transduced with indicated AAV vectors, with the top row representing untransfected cells and the bottom row representing cells transfected with *Ly6a*. Scale bar: 20 μm. For all *in vitro* analyses (C–F), an MOT of 10,000 vg per cell was used. ^∗^p ≤ 0.05; ^∗∗^p ≤ 0.01; ^∗∗∗^p ≤ 0.001; ^∗∗∗∗^p ≤ 0.0001. Statistical significance was calculated using a 2-way ANOVA with Sidak’s multiple comparisons test.

Analysis revealed no detectable transgene expression or VCN in the brain (Figures 6A and 6B) or spinal cord (Figures S11A and S11C) of mice injected with the AAV-BBB variants. Although the ability to cross the BBB was not maintained in BALB/cJ mice, the observed detargeting effect in the liver, previously observed in FRG mice, remained consistent (Figures S11B and S11D).

When it was previously observed that the AAV-PHP.B family of vectors were unable to cross the BBB in BALB/cJ mice, further investigations revealed that they exhibited a dependence on the LY6A receptor for transport into the CNS.^14^^,^^15^^,^^16^ Notably, LY6A is a receptor that is unique to certain mouse strains and most critically is not present in NHPs or humans. To investigate whether our AAV-BBB variants also use LY6A for BBB binding and crossing, we performed an *in vitro* binding assay and expression analysis using HEK293T cells, with and without *Ly6a* expression (*Ly6a* and -, respectively) (Figures 6C–6F). The results showed that the binding capacity of AAV-BBB28 and AAV-BBB31 increased by more than 100-fold in *Ly6a*-expressing cells, similar to what was reported for AAV-PHP.eB (Figure 6B). This trend was further supported by expression analysis performed by FACS, which demonstrated a significant increase in transduction efficiency (Figure 6C). Similarly, the GFP mean fluorescence intensity (MFI) was significantly higher for the AAV-BBB variants in the presence of *Ly6a* (Figure 6E). Importantly, no significant changes in binding or transduction ability were observed for AAV9 and AAV1, regardless of *Ly6a* expression (Figures 6C–6F and S12).

Because we previously observed decreased brain transduction with our rationally designed NAb-evading variants (Figure 5), we next performed the same LY6A *in vitro* binding assay to confirm whether their reduction in BBB crossing efficiency was due to a loss of LY6A binding. (Figure S12A). The results showed that the binding capacity of AAV-BBB.VR-IV was diminished compared to WT AAV-BBB, whereas for AAV-BBB.VR-VIII there is no binding with LY6A. The same trend was observed by expression analysis performed by FACS, which demonstrated a significant but lower increase in transduction efficiency for BBB.VR-IV variants, while no significant changes in transduction ability were observed for BBB.VR-VIII variants (Figures S12B and S12C).

These findings show that the enhanced binding and transduction capacity of AAV-BBB28 and AAV-BBB31 variants in the presence of LY6A are consistent with the behavior reported for AAV-PHP.eB, indicating a shared dependency on the LY6A receptor as a gateway to the brain.

## Discussion

The development of safe and effective gene delivery methods is critical to enable the advancement of gene therapies for the treatment of neurodegenerative and neurodevelopmental disorders. In our study, we performed capsid sequence shuffling based on WT AAV variants 1–12 to bioengineer novel variants capable of crossing the BBB. This approach deviates from the conventional reliance on AAV9 peptide variants, which dominate the current literature involving brain-penetrating AAV capsids. Using this method, the distinctive attributes of various AAV clades within our shuffled variants provide unique capsid properties and tropisms, while retaining BBB-crossing capability in C57BL/6J mice.

Our newly identified variants, AAV-BBB6, AAV-BBB28, and AAV-BBB31, aligned most closely with AAV1 and exhibited substantial improvement over the widely used AAV9 for delivering transgenes to the CNS via IV injection in FRG mice (C57BL/6J background). Our AAV-BBB variants also demonstrated clear neuronal tropism and regional bias for the brainstem and midbrain structures, while also exhibiting significantly reduced targeting from both mouse and human hepatocytes. This reduced liver targeting in human hepatocytes when compared to clinically relevant AAV9 was confirmed *in vivo* using the hFRG chimeric liver mouse model,^18^^,^^27^ highlighting the potential of our novel variants to minimize off-target effects in the liver compared to AAV9. Notably, the diminished transduction in human hepatocyte observed in the AAV-BBB variants was also observed with the closest homolog capsid, AAV1. This suggests that the reduced liver tropism of the AAV-BBB variants is likely attributed to the close sequence similarities shared with AAV1. This property also highlights the benefits of using bioengineering strategies centered on non-AAV9 capsids to mitigate liver targeting effects.

Moreover, through the implementation of rational design principles,^26^ we were able to further optimize AAV-BBB6 and AAV-BBB28, resulting in improved immune escape properties beyond those of AAV9. This enhancement in immune escape mechanisms is crucial for evading immune responses and maximizing the therapeutic potential of the gene therapy approach.^26^

Following further examination of the AAV-BBB variants capsid properties and binding capacities, we successfully identified the LY6A receptor as either a primary receptor or co-receptor for binding and BBB entry in mice. Interestingly, the LY6A receptor is also used for the well-known AAV9 variants AAV-PHP.eB,^14^^,^^15^^,^^16^ AAV.CAP-B10, and AAV.CAP-B22,^13^ despite the different bioengineering methods used to develop those vectors.^19^^,^^28^^,^^29^ These findings mark the first occurrence of a *de novo* receptor being identified for AAVs bioengineered using shuffle-capsid technologies. It also describes the first known instance of AAV capsids originating from different classes sharing the same receptor usage. It is interesting that despite the shared use of the same receptor for BBB transport in C57BL/6J mice, our AAV variants and AAV-PHP.eB exhibit notable differences in vector distribution throughout the CNS. Specifically, our AAV variants display a primary targeting preference for midbrain and brainstem structures, whereas AAV-PHP.eB exhibits additional targeting of the outer cortex.^28^

Only limited studies have successfully identified the receptors responsible for mediating BBB crossing of bioengineered AAV capsids, all of which are AAV-peptide insertion variants.^13^^,^^15^^,^^30^^,^^31^^,^^32^^,^^33^ Because our BBB variants lack a deliberately engineered receptor ligand that is responsible for the LY6A binding (as in the case of AAV-PHP.eB and AAV.CAP-B10), further investigation of our variants may provide important insights into the interplay between the parental capsid properties, cell surface-binding domains, and key regions/residues governing cellular tropism and BBB traversal.

Although the specific region or regions responsible for LY6A binding in our BBB variants remain unidentified, our investigation hints at the involvement of the VR-V, VR-IV, and VR-VIII loops. Across all of the top-performing AAV-BBB variants, the deletion of N498 and the substitution of T502A within the VR-V loop was conserved. In addition, we noted a decrease in LY6A binding following mutation at the VR-IV loop and the complete loss of LY6A binding following mutation at the VR-VIII loop. Importantly, VR-V, VR-IV and VR-VIII form part of the AAV capsids 3-fold symmetry axis, which is known to play an essential role in cell attachment, receptor binding, and post-entry trafficking.^31^^,^^34^ Because capsid-shuffle variants (such as our AAV-BBB variants) likely require the 3 separate regions of the 3-fold symmetry axis to facilitate receptor binding and BBB crossing, it is far more difficult to identify receptor usage and engineer *in silico* ligands for specific receptor binding, which are current emerging trend for improving the efficiency and specificity of AAV9-peptide variants.^13^^,^^35^

Although it is improbable that our BBB variants will successfully cross the BBB in NHPs or humans, given their dependence on the LY6A receptor (absent in primates), exploring the delivery of these vectors through intracerebrospinal fluid routes, such as intrathecal (IT) injection, holds promise. Injection by the IT route could offer even greater specificity to the CNS with a reduced overall dose. Notably, the BBB variants share close similarities with AAV1, which has demonstrated high efficiency in CNS transduction following IT injection in NHP models.^36^ In addition, the LY6A-binding variant AAV-PHP.eB has shown excellent performance following IT delivery in NHP models.^37^ Importantly, IT injection of AAV9 in NHP models has resulted in vector leakage into the periphery, leading to strong liver transduction.^38^^,^^39^ However, due to the enhanced liver detargeting achieved in both murine and human hepatocytes with our AAV-BBB variants, the off-target effects resulting from IT leakage is expected to be minimal. Therefore, exploring the use of IT injection for delivering our BBB variants could provide a promising avenue for achieving efficient and targeted gene delivery to the CNS, particularly if their neuronal targeting preference observed following IV injection is retained following IT administration.

Although the shared usage of murine LY6A by both our BBB variants and PHP.eB-derived variants is of scientific interest, it prompts us to reconsider the use of mouse models for the evolution of translational capsids. In contrast, the selection of capsids using the NHP preclinical model holds the potential to result in capsids with greater translational capacity than humans. Notably, two recent studies have pursued AAV capsid evolution using AAV9 peptide libraries directly in NHPs, resulting in the development of the AAV.CAP-Mac and the AAV-PAL capsid variants.^24^^,^^40^ These evolved capsids demonstrated significant improvements in CNS transduction efficiency compared to AAV9, as well as murine-selected AAV variants, when assessed in NHPs. During the evolution of the AAV-PAL variants, the authors performed a selection using the same AAV capsid library in both C57BL6/J and BALB/cJ mice, as well as juvenile cynomolgus macaques.^24^ They found that the top-performing variants were consistent between both mouse strains but did not overlap with the variants selected in macaques. This discovery highlights the importance of the animal model chosen for capsid selection and screening and how this choice can significantly affect the outcomes of novel AAV variant selection.

Another important consideration when applying NHP models to AAV capsid selection is the species and age of the NHP. Despite both Old World and New World monkeys being evolutionarily closer to *Homo sapiens* than rodents, significant evolutionary gaps exist between these primates and humans.^41^^,^^42^^,^^43^ These distinctions underscore the fact that capsids selected using NHP models may not necessarily directly translate into human use, and species-specific variations need to be taken into account. In addition, the age of the NHP can significantly affect the efficiency of AAV transductionbecause BBB permeability is known to increase during certain developmental stages.^44^^,^^45^^,^^46^ The influence of NHP species and age on AAV efficiency is well documented in the study by Chuapoco et al., following directed evolution of AAV.CAP-Mac in NHP species.^40^ Their study revealed distinct differences in vector efficiency and cellular tropism depending on the developmental stage and species of NHP, as assessed in marmosets, rhesus macaques, and green monkeys.^40^ Given these known disparities, as well as ethical and financial considerations associated with the use of a large number of NHPs, it becomes imperative to carefully design workflows that strike a balance between minimizing NHP use and ensuring the efficiency and validation of capsids using these models.

In conclusion, our study has successfully developed novel AAV-BBB variants AAV-BBB6, AAV-BBB28, and AAV-BBB31, which demonstrate superior transduction efficiency in the CNS compared to AAV9. These variants exhibit neuronal tropism, regional bias for specific brain structures, and reduced liver targeting properties. Notably, the BBB variants mark a significant milestone as the first AAV-shuffle capsids with a recognized receptor that facilitates BBB penetration. The LY6A receptor identified is also used by the previously engineered AAV-PHP.B family of AAV9 variants, highlighting the first instance of AAV capsids from two separate clades using the same receptor usage. Due to the unique structural properties of our BBB variants, developing a deeper understanding of their capsid-receptor interactions and key residues responsible for facilitating specific tissue tropisms and the BBB penetration may facilitate AAV capsids to be rationally designed for greater efficiency, specificity, and translational outcomes.

## Materials and methods

### Cell lines

Adherent HEK293T cell line (American Type Culture Collection, catalog no. CRL-3216) were maintained in DMEM (Gibco, catalog no. 11965) supplemented with 10% fetal bovine serum (FBS) (Sigma-Aldrich, catalog no. F9423), 1× penicillin-streptomycin (PS) (Gibco, catalog no. 15070), and grown in a humidified incubator at 37°C with 5% CO_2_.

The CHO cell line variants Pro5 and Lec2 were a generous gift from Dr. Grant Logan, Gene Therapy Research Unit, Children’s Medical Research Institute (CMRI), and were grown as adherent cultures in MEM-α (Gibco, catalog no. 12571) supplemented with 10% FBS and 1× PS. Cells were maintained in a humidified incubator at 37°C with 5% CO_2_.

### Animals

All of the animal care and experimental procedures were approved by the joint Animal Care and Ethics Committee of CMRI and The Children’s Hospital at Westmead.

Adult male BALB/cJ mice (6–8 weeks of age) were purchased from Australian BioResources. Mice underwent a 1-week acclimatization period to relocate to the CMRI Animal Facility and were subsequently housed in standard conditions in a 12-h light/12-h dark environment. The established colony of Fah^−/−^/Rag2^−/−^/Il2rg^−/−^ (FRG) mice at CMRI was used to breed naive recipient FRG animals.^47^ FRG mice were housed in individually ventilated cages with 2-(2-nitro-4-trifluoro-methylbenzoyl)-1,3-cyclohexanedione (NTBC) supplemented in drinking water (8 mg/mL). Female FRG mice (6–8 weeks of age) were engrafted with primary human hepatocytes (Lonza), as described previously.^18^ hFRG mice were placed on 10% NTBC before transduction with vectors and were maintained on 10% NTBC until harvest. Detailed information on the hFRG mice used in the study, including human hepatocytes used and individual estimated repopulation levels, can be found in Table S1.

### Animal injections

The viral library was injected into naive male FRG mice through the IV route (lateral tail vein) (N = 1) at 5 × 10^11^ vg in rounds 1 and 2 of selection, and at 2 × 10^11^ vg in rounds 3 and 4. For each selection round, 3 weeks post-injection, mice were euthanized following by perfusion with cold Hank’s balanced salt solution (HBSS; Thermo Fisher, catalog no. 14025092). Whole brain was then harvested and snap frozen in liquid nitrogen before being stored at −80°C.

For NGS screening of the AAV-BBB testing kit, naive male FRG (6–8 weeks old) were randomly selected and injected IV (lateral tail vein), with the indicated vectors at a dose of 1.5 × 10^10^ vg per vector (1.8 × 10^11^ total vg). Four weeks post-injection, mice were euthanized and transcardially perfused with HBSS. The brain and spinal cord were harvested for downstream analysis.

To compare vector transduction of individual AAV variants in different strains of mice, 5 × 10^11^ vg of each AAV were individually injected IV (lateral tail vein) into hFRG mice, naive male FRG (6–8 weeks old), and male BALB/cJ mice (6–8 weeks old). At 3 weeks post-injection, naive male FRG and BALB/cJ mice were euthanized and transcardially perfused with HBSS. For male FRG mice, brain, spinal cord, liver, kidney, heart, and muscle were harvested. For BALB/cJ mice, brain, spinal cord, and liver were harvested for downstream analysis. hFRG mice were euthanized 3 weeks post-injection, with 1 lobe collected for IHC analysis, and the remainder of the liver perfused by collagenase to obtain a single-cell solution amenable for FACS (performed as previously described).^48^ Human cells were isolated from the chimeric mouse/human liver using FACS as previously described.^11^ The brain and spinal cord were harvested for downstream analysis.

### Shuffle AAV capsid library generation and library selection

The AAV library was generated as previously described^49^ using AAV variants 1–12 in the parental mix (referred to as pRC-AAVLib_1–12). To move this library from a replication competent backbone into our FT platform,^19^ the pRC-AAVLib_1–12 was digested overnight alongside the pFT-spleen focus-forming visrus (SFFV) backbone with SwaI (NEB, catalog no. R0604S) and NsiI (NEB, catalog no. R0127S). Following separation on 1% (w/v) agarose gel and purification using the Zymoclean Gel DNA Recovery Kit (Zymogen, catalog no. D4001), 1.4 μg of the insert library was ligated with 1 μg of the linearized pFT-SFFV-EGFP platform backbone at 16°C for 16 h using T4 DNA ligase (NEB, catalog no. M0202). Ligation reactions were concentrated by ethanol precipitation and electroporated into SS320 electrocompetent bacteria (Lucigen, catalog no. 60512). The recovered transformants were used to inoculate 250 mL of lysogeny broth containing 10 μg/mL trimethoprim. Total FT-SFFV-EGFP-AAVLib_1–12 library plasmid was purified using the EndoFree Maxiprep Kit (Invitrogen, catalog no. A31217).

For gDNA extraction from mouse brain tissue, the standard phenol:chloroform protocol was used after Proteinase K and RNase A digestion, as previously described without modification.^17^ The *cap* sequences from the extracted DNA were amplified by PCR using the Cap_Recovery_F/R primers (Table S2) and cloned directly using the Gibson assembly into recipient plasmid as previously described.^11^ AAV capsid open reading frames (*cap*) from round 4 of library selection were cloned into a standard packaging plasmid downstream of AAV2 *rep* using the Gibson assembly. A total of 50 randomly selected clones were sent for Sanger sequencing of the capsid coding region at the Garvan Molecular Genetics facility of the Garvan Institute of Medical Research (Darlinghurst, NSW, Australia) using primers External_Seq_F/R and internal_Cap_Seq (Table S2). To allow for the visualization of parental contribution in selected capsids, the Xover online tool was used to create crossover maps (http://qpmf.rx.umaryland.edu/xover.html).^50^

### AAV viral production and titration

All of the AAV vectors were produced in adherent HEK293T cells by triple-transfection using PEI MAX (Polysciences, catalog no. 24765-1) as previously described.^51^ Briefly, triple cotransfection of the pRep2/Cap plasmid (7.5 μg per dish), the pAd5 Helper plasmid (22 μg per dish), and the transgene plasmid (7.5 μg per dish) was performed in 15-cm dishes seeded with HEK293T cells. A modified transfection protocol was used for the AAV capsid libraries. A total of 200 ng of FT-SFFV-AAVLib_1–12 library plasmid was used per dish to minimize cross-packaging (under 10%) as per our previously published protocol.^19^ In replacement of the transgene plasmid, pRep2 helper plasmids (5 μg per dish) were transfected alongside pAd5 Helper plasmid (22 μg per dish). At 3 days post-transfection, recombinant virus was harvested from the cells and media and purified using iodixanol gradient ultracentrifugation as previously described,^52^ and concentrated using Amicon Ultra-4 Centrifuge Filter Units with Ultracel-100 kDa membrane (EMD Millipore, catalog no. UFC810024). AAV preparations were quantified by ddPCR (Bio-Rad) using EvaGreen supermix (Bio-Rad, catalog no. 1864034) and following the manufacturer’s instructions using mCherry or GFP-specific primers (Table S2).

### Cloning of AAV transgene cassettes

The 3 (ss) rAAV genomes used in this study were (1) pCMV-mCherry-WPRE-BGHpA, containing the fluorescent protein mCherry under control of the ubiquitous CMV promoter; (2) pCMV-EGFP-WPRE-BGHpA, containing the fluorescent protein EGFP under the control of the CMV promoter; and (3) phSyn-mCherry-N_6_BC-WPRE, containing the fluorescent protein mCherry under the control of the human synapsin promoter and harboring a unique 6-mer BC sequence located between mCherry and WPRE to differentiate unique capsids packaging the same transgene. For the LY6A experiments, *Ly6a* expression vector was cloned into the pAAV-CMV-EGFP-WPRE-BGHpA plasmid. EcoRI-HF (NEB, catalog no. R3101S) and HindIII-HF (NEB, catalog no. R3104S) restriction enzymes were used for the plasmid backbone and the *Ly6a* cDNA synthesized as gBlocks (IDT). Both fragments were isolated, ligated with T4 ligase (NEB), and transformed in DH5α competent cells. The construct was confirmed by Sanger sequencing.

### PCR BC amplification and NGS

Isolation of DNA and RNA and cDNA synthesis was performed as described previously.^11^ Briefly, total DNA was extracted using standard phenol:chloroform methods, and RNA was extracted using the Direct-Zol kit (Zymogen, catalog no. R2062). cDNA synthesis was performed using the SuperScript IV First-Strand Synthesis System (Invitrogen, catalog no. 18091050) according to the manufacturer’s instructions; Oligo(dT)s were used for priming. A total of 500 ng of RNA were used as input for the cDNA reaction.

For amplification and recovery of the AAV BC region for NGS analysis, 100 ng of extracted total DNA or 3 μL cDNA product was amplified using 1 of 4 available forward primers (BC_F_1–4; barcoded to allow multiplexing of different samples) and a universal reverse primer (BC_R). PCR was performed using the Q5 high-fidelity DNA polymerase (NEB, catalog no. M0491L). To analyze capsid enrichment, NGS reads from the DNA and cDNA populations were normalized to the reads from the preinjection mix and displayed as a percentage of total reads.

### IHC

To prepare tissue for the analysis of native mCherry fluorescence, tissue samples were fixed with 4% (w/v in PBS) paraformaldehyde (PFA) overnight at 4°C before being cryoprotected through a sucrose gradient (10%, 20%, and 30% w/v sucrose in PBS). Tissue samples were then frozen in optimal cutting temperature (OCT) (Tissue-Tek, Sakura Finetek) and sectioned using a cryostat (Leica, catalog no. CM1950). OCT-embedded tissues were sectioned at a thickness of 50 μm for brain, 15 μm for spinal cord, 5 μm for liver, and 20 μm for all other tissues. Tissues were stained with DAPI (Invitrogen, D1306) at 1:1,000 for 5 min before imaging.

For the immunostaining of brain, sectioned tissues were first permeabilized for 10 min with 0.2% Triton X-100 in PBS before blocking at room temperature for 1 h in blocking buffer (10% normal donkey serum in 0.2% Triton X-100 in PBS). Incubation in primary antibody rabbit anti-NeuN (1:200, Abcam, catalog no. ab177487) and rabbit anti-GFAP (1:200, DAKO, catalog no. Z0334) was then performed for 20–24 h at room temperature or overnight at 4°C for primary rabbit anti-olig2 antibody (1:200, Abcam, catalog no. ab109186) in blocking buffer. Sections were washed 3 times with PBS with 0.1% Tween before incubation in goat anti-rabbit Alexa Fluor Plus 488 secondary antibody (1:500, Thermo Fisher, catalog no. A32731) or donkey anti-rabbit Alexa Fluor Plus 647 secondary antibody (1:500, Thermo Fisher, catalog no. A32795) diluted in blocking buffer for 2 h at room temperature. For immunostaining of spinal cord sections, the same protocol was used; however, only staining with NeuN was performed.

For immunostaining of chimeric liver samples, 1 lobe was collected before liver perfusion, and liver sections (5 μm) were prepared on a cryostat (Leica, catalog no. CM1950). Sections were permeabilized with 0.2% Triton X-100 in PBS and blocked in 10% rabbit serum (Sigma-Aldrich) in PBS for 30 min at room temperature. Sections were then incubated with rabbit monoclonal anti-human GAPDH antibody conjugated with Alexa Fluor 647 (1:650, Abcam, catalog no. ab215227, clone AF674) at room temperature for 2 h.

### Imaging and quantification

Images were captured and analyzed on a Zeiss Axio Imager.M1 using ZEN 2 software. For quantitative image analysis (Figure 3), images were captured on a Leica Stellaris 8 confocal microscope using a 20× objective. mCherry^+^ NeuN cells analysis was performed with CellProfiler version 4.2.5.^53^ All of the acquired images were then processed on Fiji.^54^

### Biodistribution analysis

Vector genome VCNs from mouse tissues were determined by ddPCR after the extraction of total DNA using the DNeasy Blood and Tissue Kit (QIAGEN, catalog no. 69504). The vector genome content in each tissue was determined by digesting 60 ng of DNA per reaction with EcoRI-HF (NEB, catalog no. R3101S) and quantified by a duplex ddPCR using a primer set and probe designed specifically for mCherry or GFP and mouse actin-β as a reference (primer and probe sequences can be found in Table S2).

For VCN analysis of human hepatocytes, human cells were first isolated from the chimeric mouse/human liver using FACS as previously described.^11^ gDNA was then extracted from human hepatocyte cells using the DNeasy Blood and Tissue Kit (QIAGEN). VCN analysis was performed using DNA digested with EcoRI-HF (NEB, catalog no. R3101S) (total of 6 ng per reaction). For ddPCR quantification, primers were used that specifically target the GFP gene of the transgene cassette, with human albumin as a reference gene (primer sequences can be found in Table S2).

VCN quantification was normalized to the housekeeping gene used and multiplied by 2 to achieve VCN per diploid genome.

### ELISA measurement of anti-AAV IgG-specific antibody titer in human serum

Eight human sera were assayed for reactivity to AAV9, AAV-BBB6, AAV-BBB28, and AAV-BBB31 by ELISA as described in detail previously,^27^ with no modifications.

### Model building and structure visualization

The models of AAV-BBB6, AAV-BBB28, and AAV-BBB31 variant VP3 monomers were built by uploading their individual sequences to the protein modeling server SWISS-MODEL with the structure of AAV1 (PDB: 6JCR) as the template.^55^ Monomers visualization and superposition were generated using the software UCSF ChimeraX.^56^

### IVIg neutralization assay

Capsid reactivity with human IVIg was assessed in HEK293T cells. First, 1.5 × 10^5^ HEK293T cells per well (24-well format) were seeded 24 h before transduction with capsids to be assessed (multiplicity of transduction [MOT] of 50,000 vg per cell). For the neutralization assay, vectors were diluted in DMEM solution and incubated for 1 h at 37°C with increasing doses of human IVIg serum (undiluted, 1:1, 1:2, 1:4, 1:8, 1:16, 1:32, and no IVIg control) (Intragam 10, 10 g/100 mL, CSL Behring). The individual dose mixtures of vector and IVIg were then added to the cells, with a full media change of complete DMEM performed 16 h post-transduction. Cells were incubated for a further 32 h (48 h total) before harvesting. Transduction efficiency was assayed by flow cytometry (BD FACSCanto cell analyzer, BD Bioscience) by determining the percentage of EGFP^+^ cells. All of the readouts were normalized to controls with no IVIg treatment.

### CHO cell surface binding and transduction assays

For surface binding assays, CHO cells were seeded in standard tissue culture-coated 24-well plates (3 × 10^5^/well). The following day, the plates were preincubated in complete medium at 4°C for 1 h. Respective AAV variants were applied at an MOT of 10,000 vg per cell. The plate was subsequently incubated at 4°C for 1 h. Cells were washed 3 times with cold PBS to ensure the removal of unbound AAV particles. Cells were then lysed and harvested for total DNA extraction (including AAV viral gDNA in cell surface-bound viral particles) for ddPCR analyses. The vector genomes bound were normalized to the number of cell genomes by using primers specific to CHO-β-actin (Table S2).

For transduction assays, AAV variants were added to CHO cells 24-well plates (1.5 × 10^5^/well) at an MOT of 10,000 vg per cell and incubated at 37°C. The percentage of transgene-expressing cells was determined by flow cytometry 48 h post-transduction.

### *In vitro* LY6A surface binding assays and transduction assays

For all of the LY6A experiments, HEK293T cells were seeded in 15-cm dishes. At 24 h later, cells were transfected with PEI MAX and 25 μg of *Ly6a* cDNA. At 48 h later, the cells were transferred into a 24-well format at a density of 4 × 10^5^ per well and transduced with respective AAV variants at an MOT of 10,000 vg per cell. For the surface binding assay, cells were prechilled to 4°C for 45 min and then the media was exchanged with fresh cold media containing the indicated recombinant AAV (10,000 vg per cell). The cells were washed 3 times with cold PBS1 h later, then lysed for gDNA extraction and ddPCR analyses.

For transduction assays, AAV variants were added to cells (2 × 10^5^/well) and incubated at 37°C. At 48 h post-transduction cells were harvested, and the percentage of transgene-expressing cells was determined by flow cytometry.

For immunofluorescence, HEK293T cells were seeded on poly-d-lysine–coated sterile glass coverslips (High Precision, catalog no. 0117530) and incubated with respective AAV for 48 h post-transfection in 12-well plates. Cells were then fixed in 4% PFA for 10 min at room temperature. Coverslips were permeabilized with 0.1% Triton X-100 in PBS, then blocked with 0.2% Triton X-100 in PBS containing 10% normal goat serum for 30 min, incubated in primary antibody LY6A (1:250 dilution; Invitrogen, catalog no. 14-5981-82), and diluted in blocking buffer overnight at 4°C. Coverslips were washed 3 times in PBS and then incubated with goat anti-rat Alexa Fluor Plus 647 secondary antibody (1:500, Thermo Fisher, catalog no. A21247) in 1× PBS for 90 min at room temperature followed by DAPI at 1:1,000 in PBS. Coverslips were then mounted on slides. Once dry, fluorescent microscopy images were captured on a Leica Stellaris 8 confocal microscope using a 63× objective. Acquired images were then processed using ImageJ software.

### Statistical analysis

Statistical significance was assessed using GraphPad Prism 9 software. The test used is specified in the figure legends. For all of the statistical analyses, significance is represented as ^#/^^∗^p ≤ 0.05, ^##/^^∗∗^p ≤ 0.01, ^∗∗∗^p ≤ 0.001, and ^####/^^∗∗∗∗^p ≤ 0.0001.

## Data and code availability

All of the data generated or analyzed during this study are included in the published article.

## Supplemental information

Document S1. Figures S1–S13 and Tables S1 and S2

Document S2. Article plus supplemental information
