# Supplementary material for: Development of CNS tropic AAV1-like variants with reduced liver-targeting following systemic administration in mice
Source: Mol Ther. 2024 Feb 1;32(3):818–36. doi: 10.1016/j.ymthe.2024.01.024 (PMC10928139; doi:10.1016/j.ymthe.2024.01.024)
Supplement: Document S1. Figures S1–S13 and Tables S1 and S2 [file mmc1.pdf]

YMTHE, Volume *32*

Supplemental Information

Development of CNS tropic AAV1-like variants with reduced liver-targeting following systemic administration in mice

Matthieu Drouyer, Jessica Merjane, Deborah Nazareth, Maddison Knight, Suzanne Scott, Sophia H.Y. Liao, Samantha L. Ginn, Erhua Zhu, Ian E. Alexander, and Leszek Lisowski

Supplemental Figures

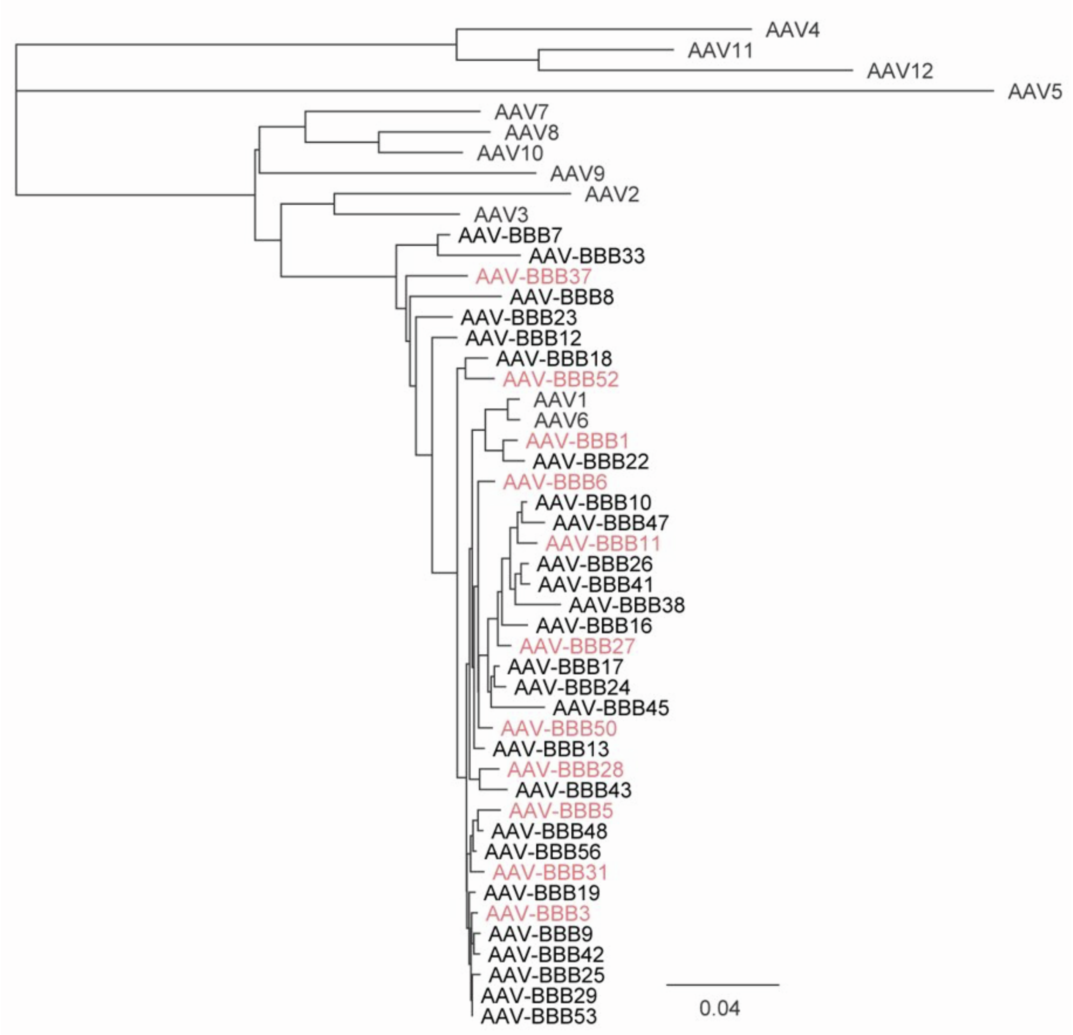

Figure S1: Dendrogram of the selected AAV-BBB clones compared to their parental capsids. Dendrogram representation is shown in scale, where branch lengths are proportional to the number of evolutionary changes (number of substitutions). Eleven node-representative candidates were chosen for further characterisation (displayed in red).

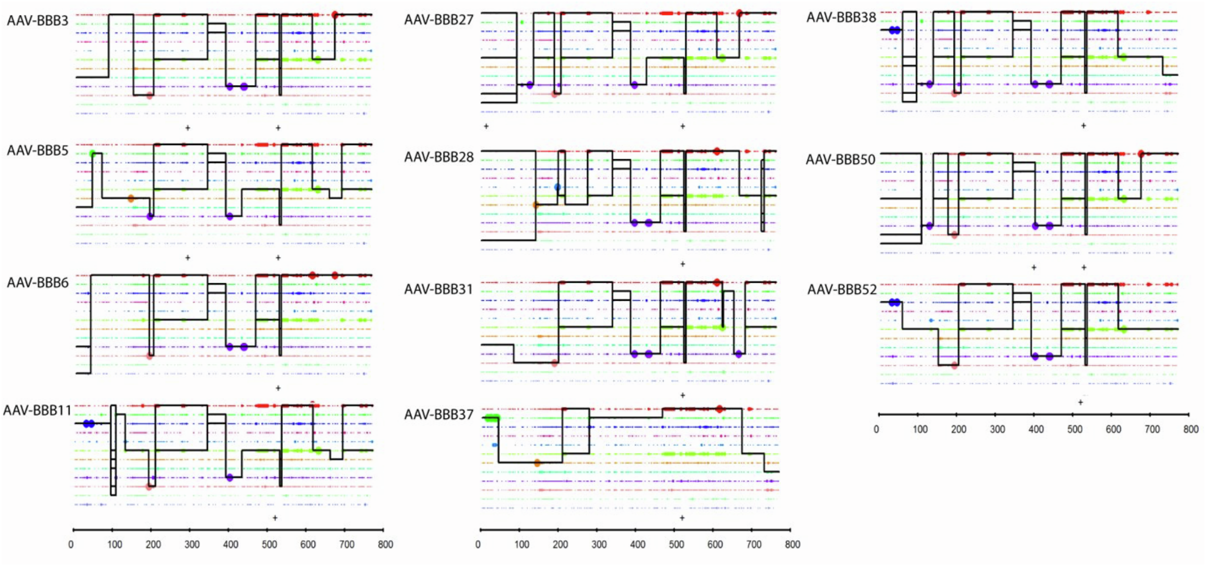

Figure S2: Parental sequence contribution of selected AAV-BBB variants selected after i.v. injection. Library parents (AAV1-12) are presented in numerical order with each represented by a unique colour. For each chimeric capsid variant, a continuous black line is used to indicate which parental strain the specific capsid region originates. Thin parallel lines connecting the crossovers indicate multiple possible parental matches due to conserved sequence regions among variants. The coloured circles represent sequence matches, with large circles indicating a 100% match of the amino acid from that specific parent at that particular position. The size of other circles are proportional to the likelihood of the amino acid at that position matching the corresponding parent. The plus sign indicates a genetic mutation that has occurred within the parental sequence space. Scale indicates amino acid position.

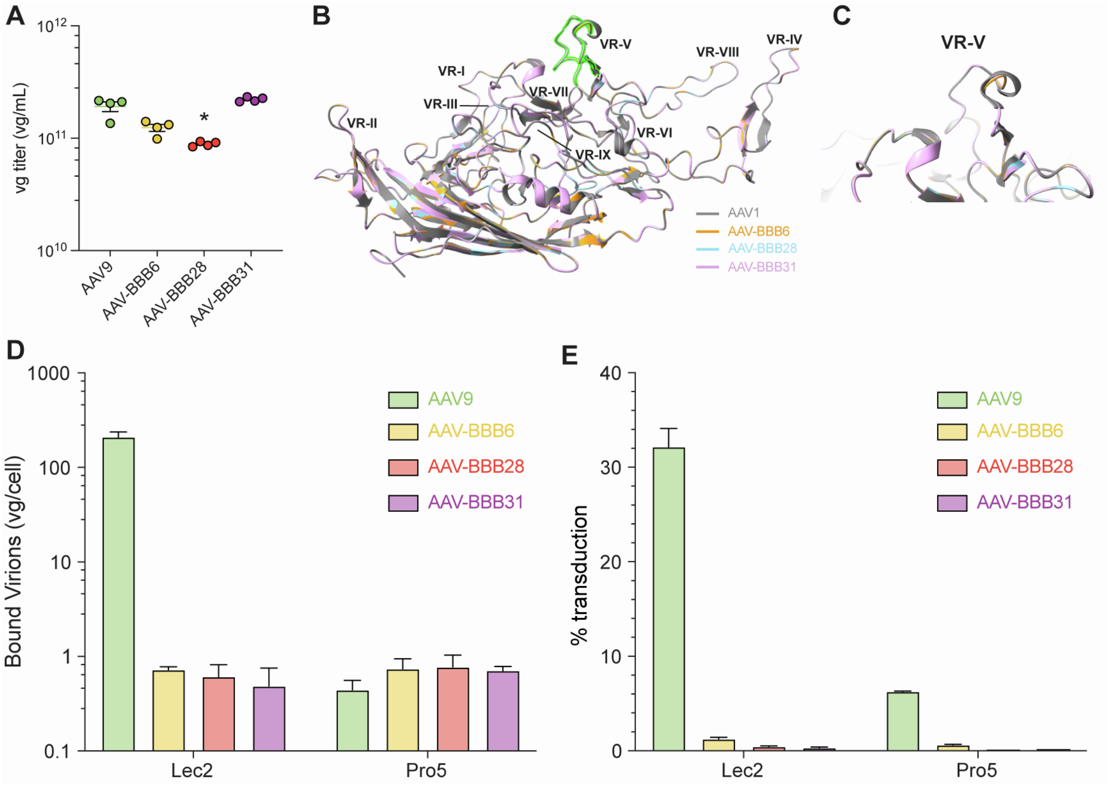

Figure S3: Characterisation of AAV-BBB variants. (A) rAAV production yield comparison. Measurement of rAAV titer following independent single-dish cell lysate production per AAV capsid (N = 4). Data is shown as total mean ± SEM. *p ≤ 0.05, Statistical significance was calculated using a one-way ANOVA nonparametric Kruskal–Wallis test and multiple comparisons with uncorrected Dunn's test. **(B)** Superposition of the VP3 monomers of AAV1 (gray), AAV-BBB6 (orange), AAV-BBB28 (blue), and AAV-BBB31 (pink), highlighting the variable regions (VR-I to -IX). As the most notable changes in structure are observed in VR-V, this region has been highlighted in green and enlarged in **(C)**. The figures were generated using the program ChimeraX. **(D)** CHO cell binding assay of AAV9 and AAV-BBB variants. Data shown as mean ± SEM. **(E)** Transduction of CHO cells as measured by percentage of GFP positive cells via FACS 48 h after vectors addition. Data shown as mean ± SEM. N = 3.

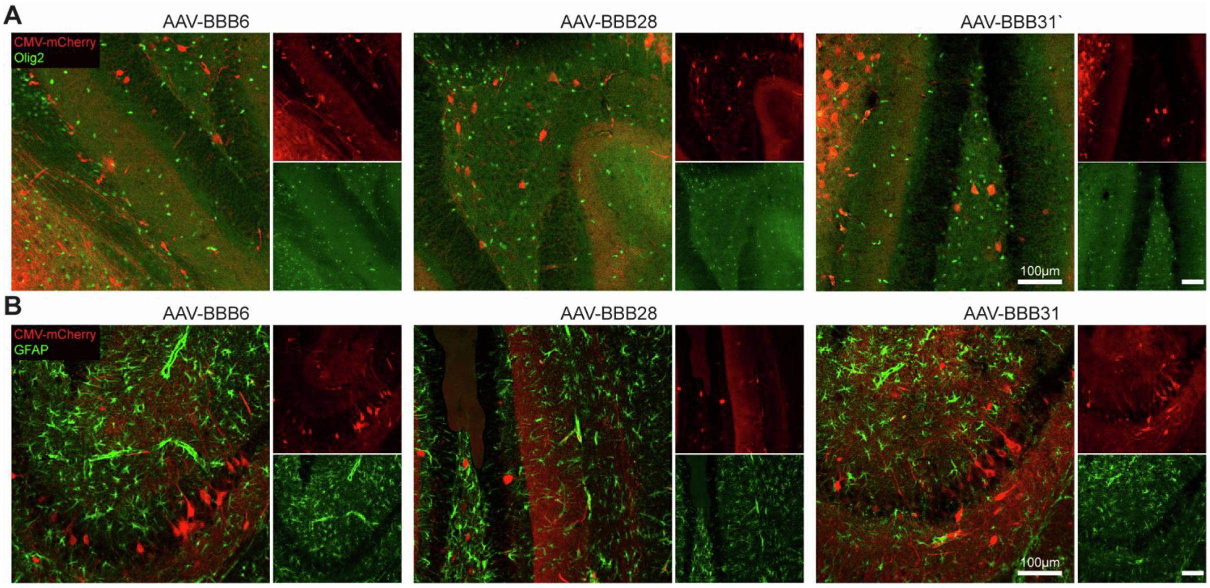

Figure S4: AAV-BBB variants do not hold preference for oligodendrocytes or astrocytes. Capsid variants AAV9, AAV-BBB6, AAV-BBB28 and AAV-BBB31 were packed with a transgene containing an mCherry reporter under the control of the ubiquitous CMV promoter. Each individual capsid variant was i.v. administered to male FRG mice with a dose of 5×10^{11} vg/animal and transgene expression was assessed by mCherry fluorescence three weeks post injection. Confocal microscopy images showing immunostaining with **(A)** Olig2 (green) for oligodendrocytes; or **(B)** GFAP (green) for astrocytes. Scale bars: 100 µm.

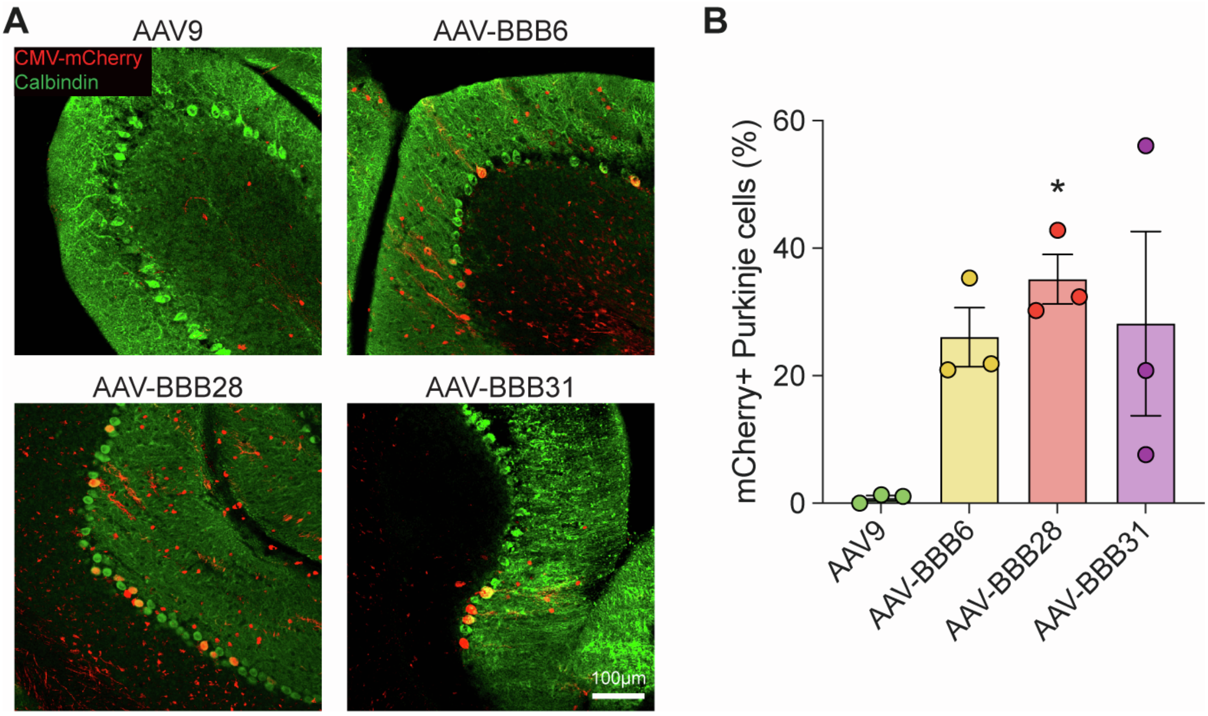

Figure S5: Enhanced targeting of Purkinje cells in the cerebellum of AAV-BBB variants compared to AAV9. Capsid variants AAV9, AAV-BBB6, AAV-BBB28 and AAV-BBB31 were packaged with a transgene containing an mCherry reporter under the control of the ubiquitous CMV promoter. Each individual capsid variant was administered i.v. to male FRG mice with a dose of 5×10^{11} vg/animal. Transgene expression was assessed by mCherry fluorescence three weeks post injection in the cerebellum. **(A)** Immunohistochemical analysis of mouse cerebellum sections stained with Calbindin for Purkinje cells (green). Scale bars: 100 µm. **(B)** Percentage of mCherry positive Purkinje cells in the brainstem, cerebellum, cortex, midbrain, hippocampus, and thalamus of mice injected with AAV9 or AAV-BBB variants (N = 3). Data is represented as the mean ± SEM. Individual data points represent the average of 3 non-overlapping images taken with a 20× objective. *$p \leq 0.05$. Statistical significance was calculated using a one-way ANOVA with Dunnetts's multiple comparisons test against AAV9 as the control.

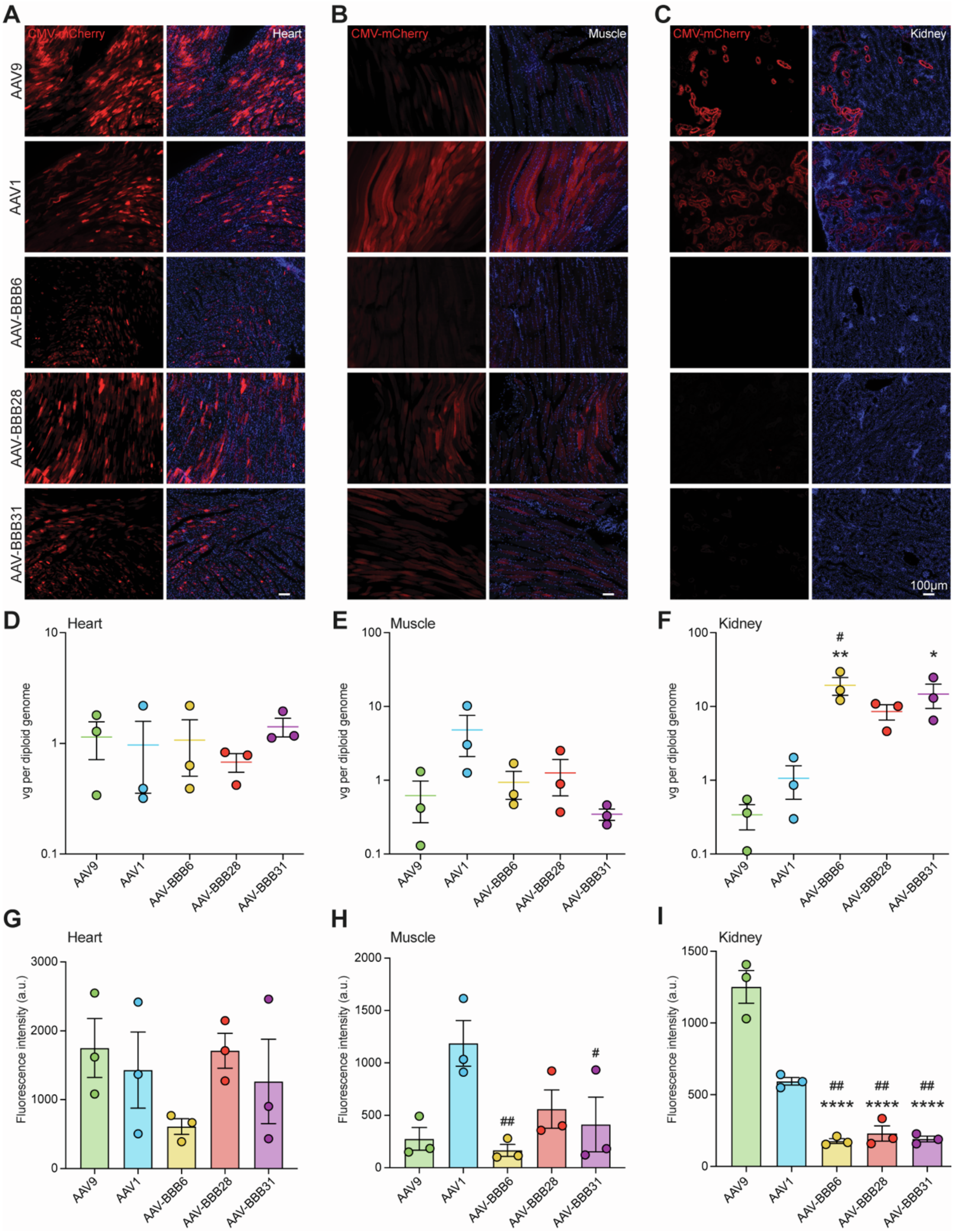

Figure S6: Biodistribution to other peripheral mouse tissues after intravenous delivery of AAV-BBB variants compared to AAV9 and parental AAV1. Capsid variants AAV9, AAV1, AAV-BBB6, AAV-BBB28 and AAV-BBB31 were packed with a transgene containing an mCherry reporter under the control of the ubiquitous CMV promoter. Each individual capsid variant was i.v. administered to male FRG mice with a dose of 5×10^{11} vg/animal. Mouse tissues were harvested three weeks post injection. Transgene expression was assessed by mCherry fluorescence in the **(A)** heart, **(B)** muscle, and **(C)** kidney. Vector copy number (VCN) was also assessed in the **(D)** heart, **(E)** muscle, and **(F)** kidney as represented by vg per diploid cell (normalized to mouse Actinβ). Quantification of the IHC fluorescence intensity was also performed for the **(G)** heart, **(H)** muscle, and **(I)** kidney. Scale bar = 100 µm. Statistical significance was calculated using a one-way ANOVA with Dunnetts's multiple comparisons test versus AAV9 (*p ≤ 0.05, **p ≤ 0.01, ****p ≤ 0.0001); or versus AAV1 ($^{\#}$p ≤ 0.05, $^{\#\#}$p ≤ 0.01)

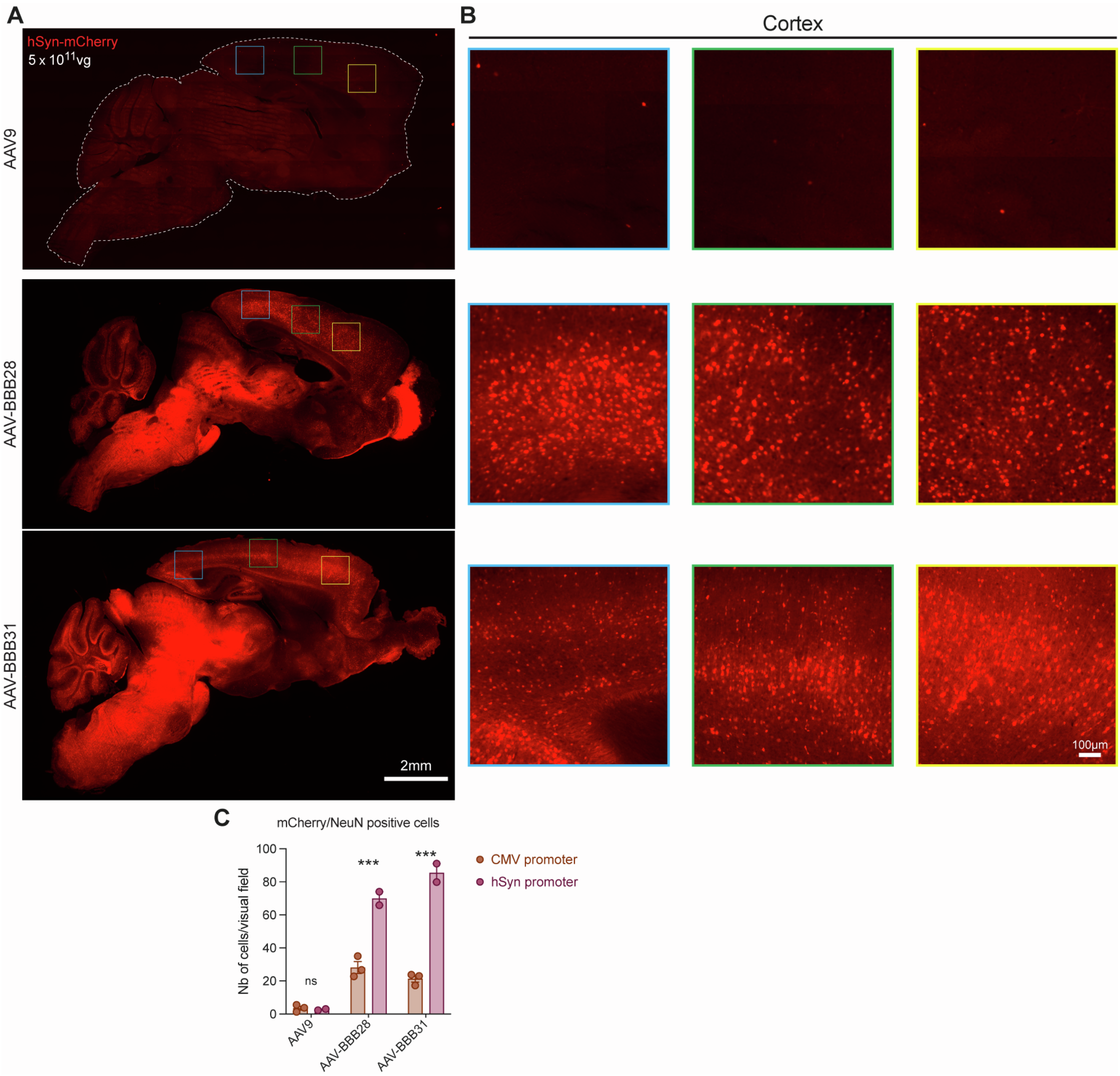

Figure S7: Utilisation of the hSyn promoter with AAV-BBB variants allows for expression in the FRG mouse brain cortex. Capsid variants AAV9, AAV-BBB28 and AAV-BBB31 were packaged with a transgene containing an mCherry reporter under the control of the hSyn promoter. Each individual capsid variant was administered i.v. to male FRG mice with a dose of 5×10^{11} vg/animal. Transgene expression was assessed by mCherry fluorescence three weeks post injection. **(A)** Immunohistochemical analysis of mouse sagittal brain sections showing mCherry expression. Scale bar: 2 mm. Coloured boxes correlating the location of the inset images displayed in **(B).** Magnified cortex region insets showing mCherry expression throughout the region (N = 2). Scale bar: 100 µm. **(C)** Quantification of the number of mCherry positive neurons in the cortex. Data is represented as the mean ± SEM. Individual data points represent the average of 3-4 non-overlapping images of the cortex region. ***$p \leq 0.001$. Statistical significance was calculated using a two-way ANOVA with Šidák's multiple comparisons test.

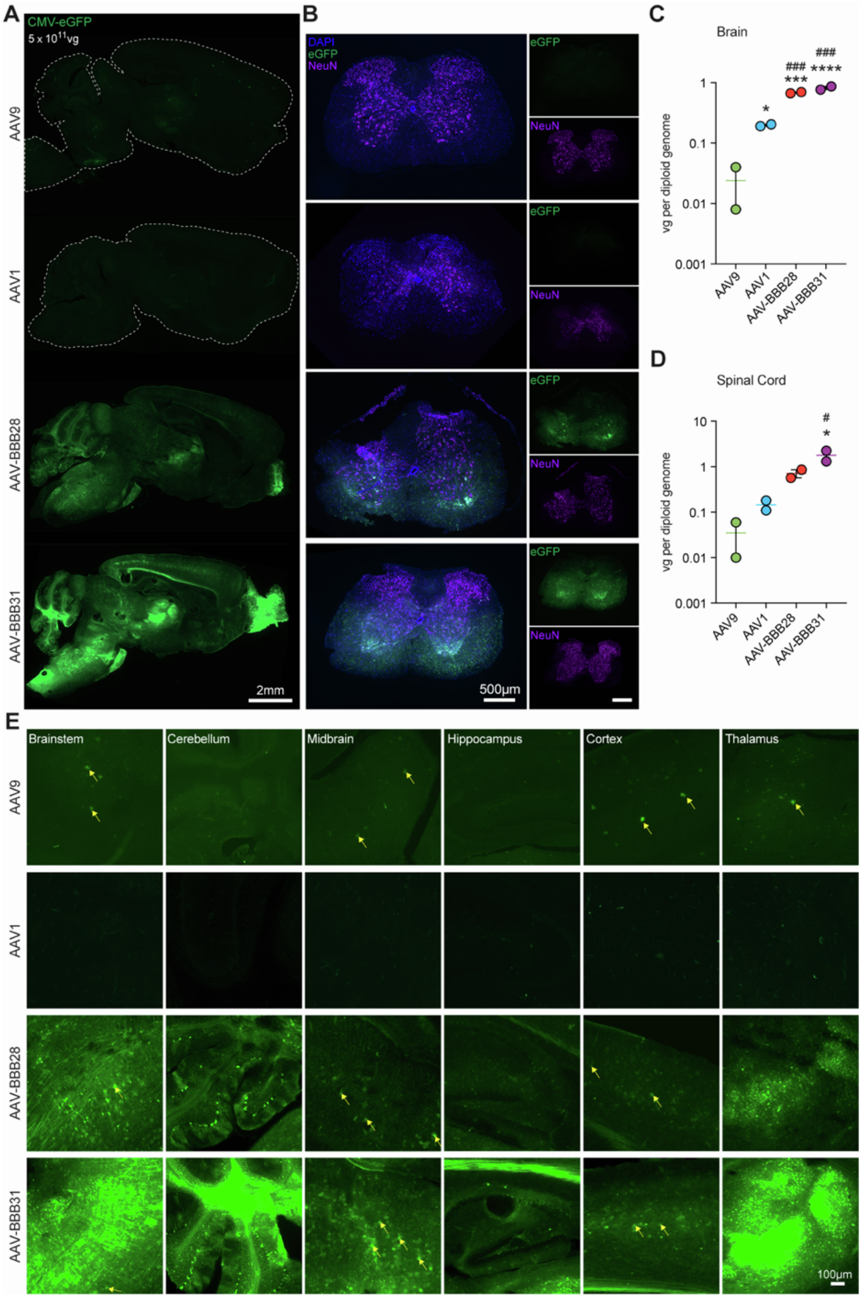

Figure S8: CNS Transduction Profile AAV-BBB28 and AAV-BBB31 in hFRG mouse CNS as compared with AAV9 and parental AAV1. Capsids were packaged with an eGFP reporter driven by a ubiquitous CMV promoter. hFRG mice were injected intravenously at a dose of 5×10^{11} vg/animal and harvested 3 weeks post injection. **(A)** eGFP expression (green) of AAV9, AAV1, AAV-BBB28 and AAV-BBB31 in hFRG mouse brain; and **(B)** spinal cord. NeuN expression is shown in purple. VCN of each variant is indicated for both **(C)** brain; and **(D)** spinal cord. Data is represented as the mean ± SEM. Statistical significance was calculated using a one-way ANOVA with Dunnetts's multiple comparisons test versus AAV9 ($^{*}p \leq 0.05$, $^{***}p \leq 0.001$, $^{****}p \leq 0.0001$) or versus AAV1 ($^{\#}p \leq 0.05$, $^{\#\#\#}p \leq 0.001$). **(E)** Region specific eGFP expression in the hFRG brain. Yellow arrows indicate examples of GFP positive astrocytes identified by morphology. Scale bar is as indicated.

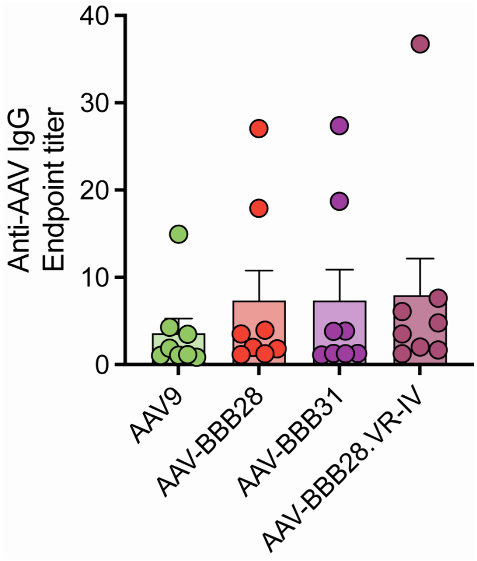

Figure S9: Prevalence of anti-AAV IgG specific antibody against AAV-BBB variants. Prevalence of anti-AAV IgG specific antibody titer in human serum (N = 8) measured by ELISA. Statistical analyses were performed by one-way ANOVA followed by Dunnett's multiple comparison test.

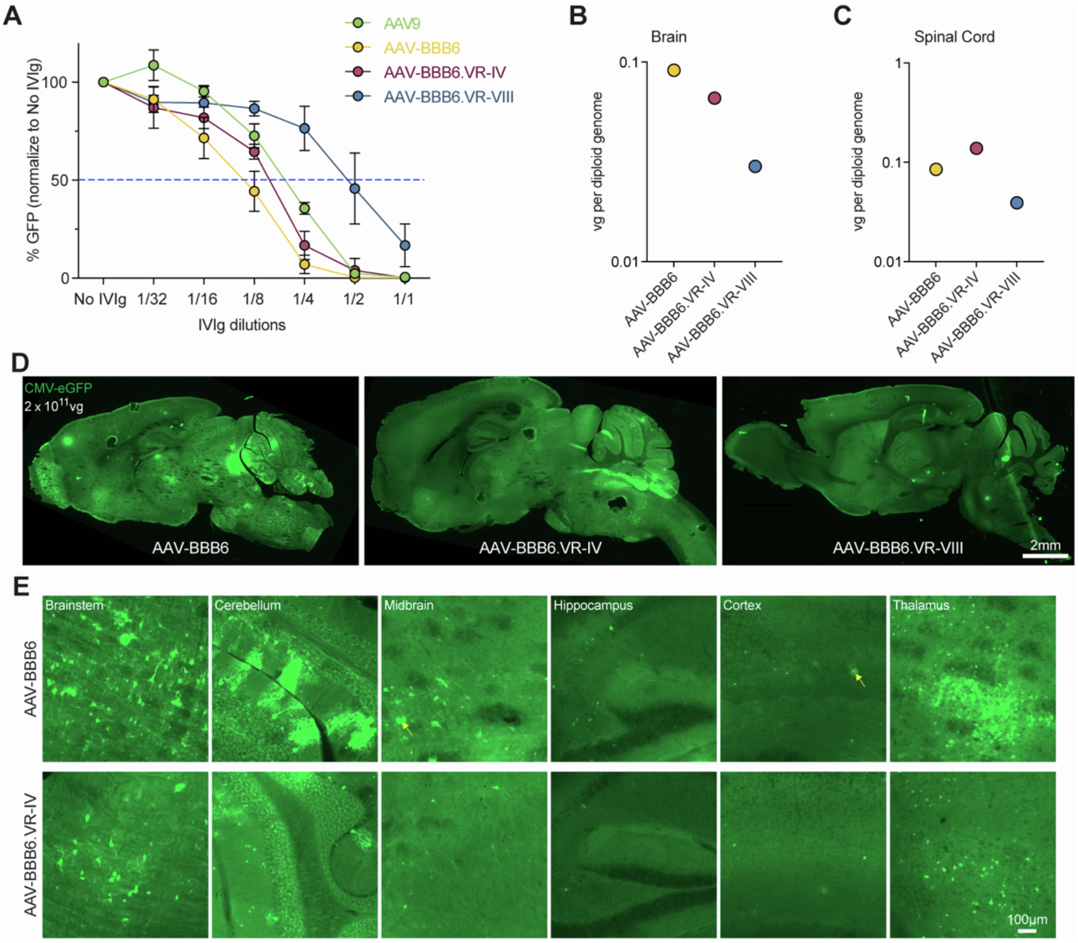

Figure S10: Enhanced IVIg resistance of AAV-BBB6 variants via semi-rational engineering. (A) Neutralisation assay of indicated AAV-BBB6 variants following pre-incubation with human IVIg prior to transduction of HEK293T cells. The percentage of GFP-positive cells 48 h after transduction was analysed by flow cytometry (N = 3 independent experiments). The dotted blue line represents IVIg-mediated inhibition of AAV transduction by 50% (IC_{50}). **(B-E)** CNS Transduction profile of AAV-BBB6.VR-IV and AAV-BBB6.VR-VIII in FRG mouse CNS as compared with wtAAV-BBB6. VCN of each variant is indicated for both **(B)** brain; and **(C)** spinal cord. **(D)** eGFP expression (green) of AAV-BBB6 variants injected i.v. in FRG mouse brain (8-week-old FRG males, 2×10^{11} total vg per mouse) harvested 3 weeks post injection. **(E)** Region specific eGFP expression in the brain. Yellow arrows indicate examples of GFP positive astrocytes identified by morphology. Scale bar is as indicated. N = 1 per group.

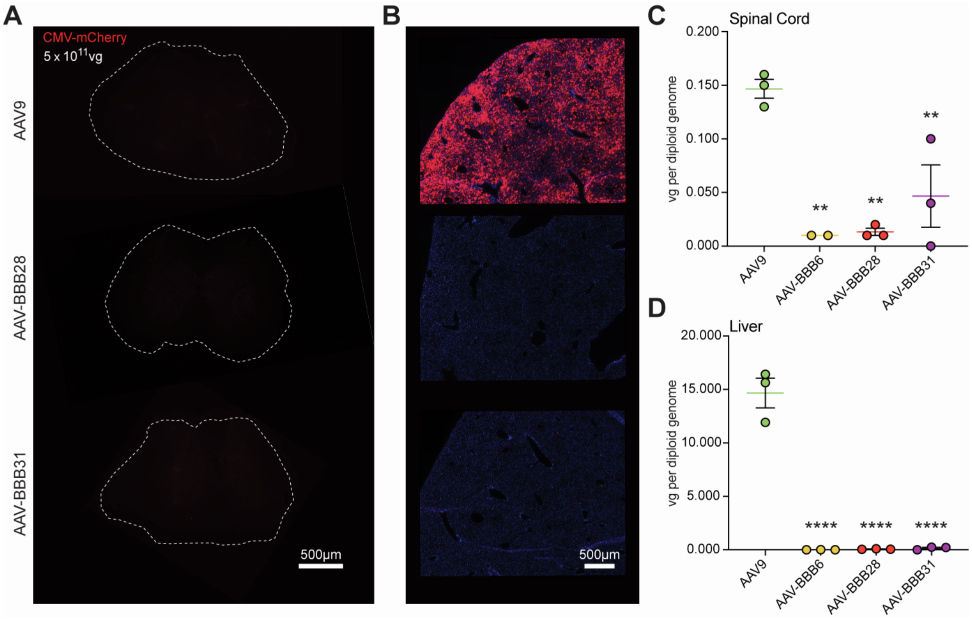

Figure S11: Transduction profile of AAV-BBB variants compared with AAV9 following systemic injection in BALB/cJ mice. IHC and VCN comparison of AAV vector performance following i.v. injection of AAV-BBB6, AAV-BBB28 or AAV-BBB31 compared to AAV9. All variants were packaged with a transgene containing an mCherry reporter under the control of the ubiquitous CMV promoter and injected at a dose of 5×10^{11} vg/animal. Mice were anaesthetised three weeks post injection, with spinal cord and liver harvested for IHC and VCN analysis. Transgene expression was assessed by mCherry fluorescence in the **(A)** spinal cord, and **(B)** liver. VCN was also assessed in the **(C)** spinal cord, and **(D)** liver as represented by vg per diploid cell (normalised to mouse Actinβ). Scale bar: 500 µm. Data is represented as the mean ± SEM. $^{**}p \leq 0.01$, $^{****}p \leq 0.0001$. Statistical significance was calculated using a one-way ANOVA with Dunnetts's multiple comparisons test against AAV9 as the control.

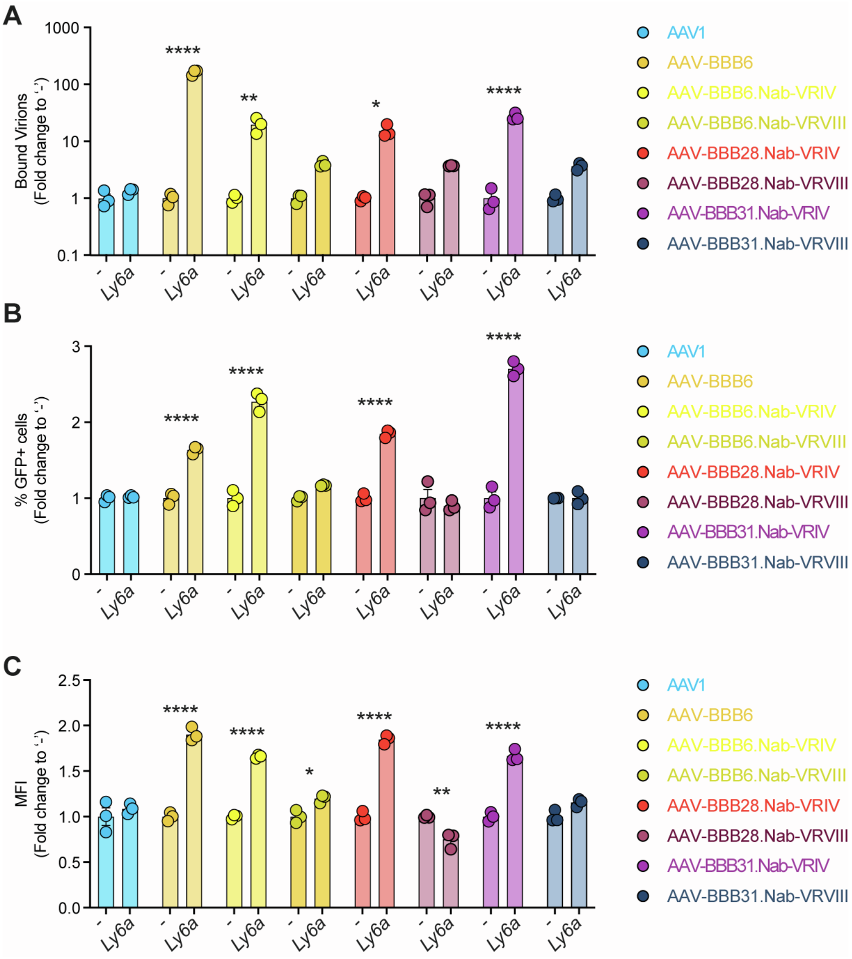

Figure S12: BBB crossing of AAV-BBB variants correlates with LY6A binding affinity. **(A)** Cell binding assay of the indicated AAV variants in HEK293T transfected with *Ly6a* or untransfected control. Binding was assessed by ddPCR of the viral genome. Data is shown as mean ± SEM. N = 3. (**B-C**) Transduction assay of indicated AAV variants as measured by FACS 48 hours post transduction. Transduction efficiency is measured by **(B)** percentage of GFP positive cells, and **(C)** mean fluorescence intensity (MFI) quantification. Data is shown as mean ± SEM. N = 3. A MOT of 1,000 vg/cell was used for AAV1; 50,000 vg/cell for AAV-BBB28.Nab-VRVIII and AAV-BBB31.Nab-VRVIII; and 10,000 vg/cell was used for all the other variants. *p ≤ 0.05, **p ≤ 0.01, ****p ≤ 0.0001. Statistical significance was calculated using a two-way ANOVA with Šidák's multiple comparisons test.

Figure S13: AAV-BBB sequences.

>AAV-BBB3 translation
MAADGYLPDWLEDNLSEGIREWWALKPGAPKPKANQQKQDDGRGLVLPGYKYLG
PFNGLDKGEPVNAADAAALEHDKAYDQQLKAGDNPYLRYNHADAEFQERLQEDTS
FGGNLGRAVFQAKKRVLEPLGLVEEGAKTAPGKKRPVEPSPQRSPDSSTGIGKKGQQ
PAKKRLNFGQTGESESVPDPQPLGEPPATPAAVGPTTMASGGGAPMADNNEGADGV
GNASGNWHCDSTWLGDRVITTSTRTWALPTYNNHLYKQISSASTGANDNHYFGYST
PWGYFDFNRFHCHFSPRDWQRLINNNWGFRPKRLNFKLFNIQVKEVTQNDGTTTIA
NNLTSTVQVFTDSEYQLPYVLGSAHQGCLPPFPADVFMIPQYGYLTLNDGSQAVGRS
SFYCLEYFPSQMLRTGNNFQFSYEFENVPFHSSYAHSQSLDRLMNPLIDQYLYYLNR
TQNQSGSAQNKDLLFSRGSPAGMSVQPKNWLPGPCYRQQRVSKTKTDNNSNFAWT
GASKYNLNGRESIINPGTAMASHKDDEDKFFPMSGVMIFGKESAGASNTALDNVMIT
DEEEIKATNPVATERFGTVAVNLQSSSTDPATGDVHVMGALPGMVWQDRDVYLQG
PIWAKIPHTDGHFHPSPLMGGFGLKNPPPQILIKNTPVPANPPAEFSATKFASFITQYST
GQVSVEIEWELQKENSKRWNPEVQYTSNYAKSANVDFTVDNNGLYTEPRPIGTRYL
TRPL

>AAV-BBB5 translation
MAADGYLPDWLEDNLSEGIREWWALKPGAPKPKANQQKQDDSRGLVLPGYKYLG
PFNGLDKGEPVNAADAAALEHDKAYDQQLKAGDNPYLRYNHADAEFQERLQEDTS
FGGNLGRAVFQAKKRVLEPLGLVEEGAKTAPAKKRPVEPSPQRSPDSSTGIGKKGQQ
PARKRLNFGQTGDTESVPDPQPLGEPPATPAAVGPTTMASGGGAPMADNNEGADGV
GNASGNWHCDSTWLGDRVITTSTRTWALPTYNNHLYKQISSASTGANDNHYFGYST
PWGYFDFNRFHCHFSPRDWQRLINNNWGFRPKRLNFKLFNIQVKEVTQNDGTTTIA
NNLTSTVQVFTDSEYQLPYVLGSAHQGCLPPFPADVFMIPQYGYLTLNDGSQAVGRS
SFYCLEYFPSQMLRTGNNFTFSYTFEDVPFHSSYAHSQSLDRLMNPLIDQYLYYLNRT
QNQSGSAQNKDLLFSRGSPAGMSVQPKNWLPGPCYRQQRVSKTKTDNNSNFAWTG
ASKYNLNGRESIINPGTAMASHKDDEDKFFPMSGVMIFGKESAGASNTALDNVMITD
EEEIKATNPVATERFGTVAVNLQSSSTDPATGDVHVMGALPGMVWQDRDVYLQGPI
WAKIPHTDGNFHPSPLMGGFGLKHPPPQILIKNTPVPANPPAEFSATKFASFITQYSTG
QVSVEIEWELQKENSKRWNPEVQYTSNYAKSANVDFTVDNNGLYTEPRPIGTRYLT
RPL

>AAV-BBB6 translation
MAADGYLPDWLEDNLSEGIREWWALKPGAPQPKANQQKQDDGRGLVLPGYKYLG
PFNGLDKGEPVNAADAAALEHDKAYDQQLKAGDNPYLRYNHADAEFQERLQEDTS
FGGNLGRAVFQAKKRVLEPLGLVEEGAKTAPGKKRPVEQSPQEPDSSSGIGKTGQQP
AKKRLNFGQTGESESVPDPQPLGEPPATPAAVGPTTMASGGGAPMADNNEGADGV
GNASGNWHCDSTWLGDRVITTSTRTWALPTYNNHLYKQISSASTGASNDNHYFGYS
TPWGYFDFNRFHCHFSPRDWQRLINNNWGFRPKRLNFKLFNIQVKEVTQNDGTTTIA
NNLTSTVQVFTDSEYQLPYVLGSAHQGCLPPFPADVFMIPQYGYLTLNDGSQAVGRS
SFYCLEYFPSQMLRTGNNFQFSYEFENVPFHSSYAHSQSLDRLMNPLIDQYLYYLNR
TQNQSGSAQNKDLLFSRGSPAGMSVQPKNWLPGPCYRQQRVSKTKTDNNSNFAWT
GASKYNLNGRESIINPGTAMASHKDDEDKFFPMSGVMIFGKESAGASNTALDNVMIT
DEEEIKATNPVATERFGTVAVNFQSSSTDPATGDVHAMGALPGMVWQDRDVYLQG
PIWAKIPHTDGHFHPSPLMGGFGLKNPPPQILIKNTPVPANPPAEFSATKFASFITQYST
GQVSVEIEWELQKENSKRWNPEVQYTSNYAKSANVDFTVDNNGLYTEPRPIGTRYL
TRPL

>AAV-BBB11 translation
MAAGGCLPDWLEDNLSEGIREWWALKPGVPQPKANQQHQDNRRGLVLPGYKYLG
PGNGLDKGEPVNEADAAALEHDKAYDQQLKAGDNPYLRYNHADAEFQERLKEDTS
FGGNLGRAVFQAKKRVLEPFGLVEEGAKTAPGKKRPVEQSPQEPDSSSGIGKTGQQP
AKKRLNFGQTGESESVPDPQPIGEPPATPAAVGPTTMASGGGAPMADNNEGADGVG
NASGNWHCDSTWLGDRVITTSTRTWALPTYNNHLYKQISSASTGASNDNHYFGYST
PWGYFDFNRFHCHFSPRDWQRLINNNWGFRPKRLNFKLFNIQVKEVTQNDGTTTIA
NNLTSTVQVFTDSEYQLPYVLGSAHQGCLPPFPADVFMIPQYGYLTLNDGSQAVGRS
SFYCLEYFPSQMLRTGNNFTFSYTFEDVPFHSSYAHSQSLDRLMNPLIDQYLYYLNRT
QNQSGSAQNKDLLFSRGSPAGMSVQPKNWLPGPCYRQQRVSKTKTDNNSNFAWTG
ASKYNLNGRESIINPGTAMASHKDDEDKFFPMSGVMIFGKESAGASNTALDNVMITD
EEEIKATNPVATERFGTVAVNLQSSSTDPATGDVHVMGALPGMVWQDRDVYLQGPI
WAKIPHTDGNFHPSPLMGGFGLKHPPPQILIKNTPVPANPPAEFSATKFASFITQYSTG
QVSVEIEWELQKENSKRWNPEVQYTSNYAKSANVDFTVDNNGLYTEPRPIGTRYLT
RPL

>AAV-BBB27 translation
MAADGCLPDWLEDNLSEGIREWWDLKPGAPKPKANQQKQDDGRGLVLPGYKYLG
PFNGLDKGEPVNAADAAALEHDKAYDQQLKAGDNPYLKYNHADAEFQERLKEDTS
FGGNLGRAVFQAKKRLLEPLGLVEEGAKTAPGKKRPVEQSPQEPDSSSGIGKTGQQP
AKKRLNFGQTGESESVPDPQPIGEPPATPAAVGPTTMASGGGAPMADNNEGADGVG
NASGNWHCDSTWLGDRVITTSTRTWALPTYNNHLYKQISSASTGASNDNHYFGYST
PWGYFDFNRFHCHFSPRDWQRLINNNWGFRPKRLNFKLFNIQVKEVTQNDGTTTIA
NNLTSTVQVFTDSEYQLPYVLGSAHQGCLPPFPADVFMIPQYGYLTLNDGSQAVGRS
SFYCLEYFPSQMLRTGNNFTFSYTFEDVPFHSSYAHSQSLDRLMNPLIDQYLYYLNRT
QNQSGSAQNKDLLFSRGSPAGMSVQPKNWLPGPCYRQQRVSKTKTDNNSNFAWTG
ASKYNLNGRESIINPGTAMASHKDDEDKFFPMSGVMIFGKESAGASNTALDNVMITD
EEEIKATNPVATERFGTVAVNLQSSSTDPATGDVHVMGALPGMVWQDRDVYLQGPI
WAKIPHTDGHFHPSPLMGGFGLKNPPPQILIKNTPVPANPPAEFSATKFASFITQYSTG
QVSVEIEWELQKENSKRWNPEVQYTSNYAKSANVDFTVDNNGLYTEPRPIGTRYLT
RPL

>AAV-BBB28 translation
MAADGYLPDWLEDNLSEGIREWWDLKPGAPKPKANQQKQDDGRGLVLPGYKYLG
PFNGLDKGEPVNAADAAALEHDKAYDQQLKAGDNPYLRYNHADAEFQERLQEDTS
FGGNLGRAVFQAKKRVLEPLGLVEEGAKTAPAKKRPVEPSPQRSPDSSTGIGKKGQQ
PARKRLNFGQTGDSESVPDSQPLGEPPATPAAVGPTTMAAGGGAPMADNNEGADG
VGNASGNWHCDSTWLGDRVITTSTRTWALPTYNNHLYKQISSASTGASNDNHYFGY
STPWGYFDFNRFHCHFSPRDWQRLINNNWGFRPKRLNFKLFNIQVKEVTQNDGTTTI
ANNLTSTVQVFTDSEYQLPYVLGSAHQGCLPPFPADVFMIPQYGYLTLNDGSQAVG
RSSFYCLEYFPSQMLRTGNNFQFSYEFENVPFHSSYAHSQSLDRLMNPLIDQYLYYLN
RTQNQSGSAQNKDLLFSRGSPAGMSVQPKNWLPGPCYRQQRVSKTKTDNNSNFAW
TGASKYNLNGRESIINPGTAMASHKDDEDKFFPMSGVMIFGKESAGASNTALDNVMI
TDEEEIKATNPVATERFGTVAVNFQSSSTDPATGDVHAMGALPGMVWQDRDVYLQ
GPIWAKIPHTDGHFHPSPLMGGFGLKHPPPQILIKNTPVPANPPAEFSATKFASFITQYS
TGQVSVEIEWELQKENSKRWNPEIQYTSNYAKSANVDFTVDNNGLYTEPRPIGTRYL
TRPL

>AAV-BBB31 translation

MAADGYLPDWLEDNLSEGIREWWALKPGAPKPKANQQKQDDGRGLVLPGYKYLG
PFNGLDKGEPVNAADAAALEHDKAYDQQLKAGDNPYLRYNHADAEFQERLQEDTS
FGGNLGRAVFQAKKRVLEPLGLVEEAAKTAPGKKRPVEPSPQRSPDSSTGIGKKGQQ
PAKKRLNFGQTGESESVPDPQPLGEPPATPAAVGPTTMASGGGAPMADNNEGADGV
GNASGNWHCDSTWLGDRVITTSTRTWALPTYNNHLYKQISSASTGASNDNHYFGYS
TPWGYFDFNRFHCHFSPRDWQRLINNNWGFRPKRLNFKLFNIQVKEVTQNDGTTTIA
NNLTSTVQVFTDSEYQLPYVLGSAHQGCLPPFPADVFMIPQYGYLTLNDGSQAVGRS
SFYCLEYFPSQMLRTGNNFQFSYEFENVPFHSSYAHSQSLDRLMNPLIDQYLYYLNR
TQNQSGSAQNKDLLFSRGSPAGMSVQPKNWLPGPCYRQQRVSKTKTDNNSNFAWT
GASKYNLNGRESIINPGTAMASHKDDEDKFFPMSGVMIFGKESAGASNTALDNVMIT
DEEEIKATNPVATERFGTVAVNFQSSSTDPATGDVHVMGVLPGMVWQDRDVYLQG
PIWAKIPHTDGNFHPSPLMGGFGMKHPPPQILIKNTPVPANPPAEFSATKFASFITQYS
TGQVSVEIEWELQKENSKRWNPEVQYTSNYAKSANVDFTVDNNGLYTEPRPIGTRY
LTRPL

>AAV-BBB37 translation
MAADGYLPDWLEDTLSEGIRQWWKLKPGPPPPKPAERHKDDGRGLVLPGYKYLGP
FNGLDKGEPVNAADAAALEHDKAYDQQLKAGDNPYLRYNHADAEFQERLQEDTSF
GGNLGRAVFQAKKRVLEPLGLVEEGAKTAPAKKRPVEPSPQRSPDSSTGIGKKGQQP
ARKRLNFGQTGDSESVPDPQPLGEPPATPAAVGPTTMASGGGAPMADNNEGADGV
GNASGNWHCDSTWLGDRVITTSTRTWALPTYNNHLYKQISSQSGASNDNHYFGYST
PWGYFDFNRFHCHFSPRDWQRLINNNWGFRPKRLNFKLFNIQVKEVTQNDGTTTIA
NNLTSTVQVFTDSEYQLPYVLGSAHQGCLPPFPADVFMVPQYGYLTLNNGSQAVGR
SSFYCLEYFPSQMLRTGNNFTFSYTFEDVPFHSSYAHSQSLDRLMNPLIDQYLYYLNR
TQNQSGSAQNKDLLFSRGSPAGMSVQPKNWLPGPCYRQQRVSKTKTDNNNSNFTW
TGASKYNLNGRESIINPGTAMASHKDDEDKFFPMSGVMIFGKESAGASNTALDNVMI
TDEEEIKATNPVATERFGTVAVNFQSSSTDPATGDVHAMGALPGMVWQDRDVYLQ
GPIWAKIPHTDGHFHPSPLMGGFGLKHPPPQILIKNTPVPANPPAEFSATKFASFITQYS
TGQVSVEIEWELQKENSKRWNPEIQYTSNYYKSTSVDFAVNTEGVYSEPRPIGTRYL
TRNL

>AAV-BBB38 translation
MAADGYLPDWLEDNLSEGIREWWALKPGVPQPKANQQHQDNRRGLVLPGYKYLG
PFNGLDKGEPVNAADAAALEHDKAYDQQLKAGDNPYLKYNHADAEFQERLKEDTS
FGGNLGRAVFQAKKRLLEPLGLVEEGAKTAPGKKRPVEQSPQEPDSSSGIGKTGQQP
AKKRLNFGQTGESESVPDPQPIGEPPATPAAVGPTTMASGGGAPMADNNEGADGVG
NASGNWHCDSTWLGDRVITTSTRTWALPTYNNHLYKQISSASTGASNDNHYFGYST
PWGYFDFNRFHCHFSPRDWQRLINNNWGFRPKRLNFKLFNIQVKEVTQNDGTTTIA
NNLTSTVQVFTDSEYQLPYVLGSAHQGCLPPFPADVFMIPQYGYLTLNDGSQAVGRS
SFYCLEYFPSQMLRTGNNFQFSYEFENVPFHSSYAHSQSLDRLMNPLIDQYLYYLNR
TQNQSGSAQNKDLLFSRGSPAGMSVQPKNWLPGPCYRQQRVSKTKTDNNSNFAWT
GASKYNLNGRESIINPGTAMASHKDDEDKFFPMSGVMIFGKESAGASNTALDNVMIT
DEEEIKATNPVATERFGTVAVNLQSSSTDPATGDVHVMGALPGMVWQDRDVYLQG
PIWAKIPHTDGHFHPSPLMGGFGLKHPPPQILIKNTPVPANPPAEFSATKFASFITQYST
GQVSVEIEWELQKENSKRWNPEIQYTSNYYKSTSVDFAVNTEGVYSEPRPIGTRYLT
RNL

>AAV-BBB50 translation

MAADGYLPDWLEDNLSEGIREWWDLKPGAPKPKANQQKQDDGRGLVLPGYKYLG
PFNGLDKGEPVNAADAAALEHDKAYDQQLKAGDNPYLRYNHADAEFQERLKEDTS
FGGNLGRAVFQAKKRLLEPLGLVEEGAKTAPGKKRPVEQSPQEPDSSSGIGKKGQQP
AKKRLNFGQTGESESVPDPQPLGEPPATPAAVGPTTMASGGGAPMADNNEGADGV
GNASGNWHCDSTWLGDRVITTSTRTWALPTYNNHLYKQISSASTGASNDNHYFGYS
TPWGYFDFNRFHCHFSPRDWQRLINNNWGFRPKRLNFKLFNIQVKEVTQNDGTTTIA
NNLTSTVQVFTDSEYQLPYVLGSAHQGCLPPFPADVFMNPQYGYLTLNDGSQAVGR
SSFYCLEYFPSQMLRTGNNFQFSYEFENVPFHSSYAHSQSLDRLMNPLIDQYLYYLNR
TQNQSGSAQNKDLLFSRGSPAGMSVQPKNWLPGPCYRQQRVSKTKTDNNSNFAWT
GASKYNLNGRESIINPGTAMASHKDDEDKFFPMSGVMIFGKESAGASNTALDNVMIT
DEEEIKATNPVATERFGTVAVNLQSSSTDPATGDVHVMGALPGMVWQDRDVYLQG
PIWAKIPHTDGHFHPSPLMGGFGLKNPPPQILIKNTPVPANPPAEFSATKFASFITQYST
GQVSVEIEWELQKENSKRWNPEVQYTSNYAKSANVDFTVDNNGLYTEPRPIGTRYL
TRPL

>AAV-BBB52 translation
MAADGYLPDWLEDNLSEGIREWWALKPGVPQPKANQQHQDNRRGLVLPGYKYLG
PFNGLDKGEPVNAADAAALEHDKAYDQQLKAGDNPYLRYNHADAEFQERLQEDTS
FGGNLGRAVFQAKKRVLEPFGLVEEGAKTAPGKKRPVEPSPQRSPDSSTGIGKKGQQ
PAKKRLNFGQTGESESVPDPQPLGEPPATPAAVGPTTMASGGGAPMADNNEGADGV
GNASGNWHCDSTWLGDRVITTSTRTWALPTYNNHLYKQISSASTGASNDNHYFGYS
TPWGYFDFNRFHCHFSPRDWQRLINNNWGFRPKRLNFKLFNIQVKEVTQNDGTTTIA
NNLTSTVQVFTDSEYQLPYVLGSAHQGCLPPFPADVFMIPQYGYLTLNDGSQAVGRS
SFYCLEYFPSQMLRTGNNFQFSYEFENVPFHSSYAHSQSLDRLMNPLIDQYLYYLNR
TQNQSGSAQNKDLLFSRGSPAGMSVQPKNWLPGPCYRQQRVSKTKTDKQQQLAWT
GASKYNLNGRESIINPGTAMASHKDDEDKFFPMSGVMIFGKESAGASNTALDNVMIT
DEEEIKATNPVATERFGTVAVNLQSSSTDPATGDVHVMGALPGMVWQDRDVYLQG
PIWAKIPHTDGHFHPSPLMGGFGLKHPPPQILIKNTPVPANPPAEFSATKFASFITQYST
GQVSVEIEWELQKENSKRWNPEVQYTSNYAKSANVDFTVDNNGLYTEPRPIGTRYL
TRPL

Table S1. Information of primary human hepatocyte donors used to engraft FRG mice.

Sex	Internal ID	Albumin Level [mg/ml]	Vector	Cell origin	Replacement Index
**Female**	648	9.78	AAV9	P0 normal human hepatocytes (WT-AU#1)	64.1%
**Female**	155	14.80	AAV9	Lonza #HUM181141	55%
**Female**	335	20.312	AAV-BBB28	Lonza #HUM181971	75.1%
**Female**	170	12.798	AAV-BBB28	Lonza #HUM181141	80.1%
**Female**	358	19.916	AAV-BBB31	Lonza #HUM181971	67.4%
**Female**	162	15.853	AAV-BBB31	Lonza #HUM181141	86.1%
**Female**	61	17.144	AAV1	Lonza #HUM181971	87.2%
**Female**	62	14.808	AAV1	Lonza #HUM181971	76.7%

Table S2. Oligonucleotide and probe sequences.

**Primer**	**Sequence (5' – 3')**
**Cap_Recovery_F**	CCCTGCAGACAATGCGAGAGAATGAATCAGAATTCAAATATCTGC
**Cap_Recovery_R**	ATGCATATGGAAACTAGATAAGAAAGAAATACG
**External_Seq_F**	TGTGGATTTGGATGACTGC
**External_Seq_R**	GACCAAAGTTCAACTGAAACG
**Internal_Cap_Seq**	GTTCTTGATGAGAATCTGTGGAGGAGG
**mCherry_F**	cactacgacgctgaggtcaa
**mCherry_R**	gtgggaggtgatgtccaact
**GFP_F**	TCAAGATCCGCCACAACATC
**GFP_R**	TTCTCGTTGGGGTCTTTGCT
**BC_F_1**	GTTCAaacagtacgaacgcgccgagggc
**BC_F_2**	GTCATaacagtacgaacgcgccgagggc
**BC_F_3**	CTGTAaacagtacgaacgcgccgagggc
**BC_F_4**	GTATTaacagtacgaacgcgccgagggc
**BC_R**	CAACATAGTTAAGAATACCAGTCAATCTTTCACAAATTTTGTAATCCAGAGG
**mActinβ_F**	CCTGTATGCCTCTGGTCGTA
**mActinβ_R**	CCTCGTAGATGGGCACAGT
**hAlbumin_F**	TGCTGTCATCTCTTGTGGGCTG
**hAlbumin_R**	AACTCATGGGAGCTGCTGGTTC
**CHO-βactin_F**	CCATGTACGTAGCCATTCAGG
**CHO-βactin_R**	CATGAGGGAGAGCGTAGCC
**Probe**	**Sequence (5' – 3')**
**mCherry (FAM)**	ACCACCTACAAGGCCAAGAAGCC
**GFP (FAM)**	CGACCACTACCAGCAGAACA
**mActinβ (HEX)**	ACAGGCATTGTGATGGACTCCGG
